# Organ-specific responses during acclimation of mycorrhizal and non-mycorrhizal tomato plants to a mild water stress reveal differential local and systemic hormonal and nutritional adjustments

**DOI:** 10.1007/s00425-023-04192-2

**Published:** 2023-06-27

**Authors:** David H. Fresno, Sergi Munné-Bosch

**Affiliations:** 1 Department of Evolutionary Biology, Ecology and Environmental Sciences, Faculty of Biology, University of Barcelona, 08028 Barcelona, Spain; 2 Institute of Nutrition and Food Safety (INSA), Faculty of Biology, University of Barcelona, 08028 Barcelona, Spain

**Keywords:** Arbuscular mycorrhiza, Nutrients, Phytohormones, *Solanum lycopersicum*, *Rhizoglomus irregulare*, Water deficit

## Abstract

**Main conclusion:**

**Tomato plant acclimation to a mild water stress implied tissue-specific hormonal and nutrient adjustments, being the root one of the main modulators of this response.**

**Abstract:**

Phytohormones are key regulators of plant acclimation to water stress. However, it is not yet clear if these hormonal responses follow specific patterns depending on the plant tissue. In this study, we evaluated the organ-specific physiological and hormonal responses to a 14 day-long mild water stress in tomato plants (*Solanum lycopersicum* cv. Moneymaker) in the presence or absence of the arbuscular mycorrhizal fungus *Rhizoglomus irregulare*, a frequently used microorganism in agriculture. Several physiological, production, and nutritional parameters were evaluated throughout the experiments. Additionally, endogenous hormone levels in roots, leaves, and fruits at different developmental stages were quantified by ultrahigh-performance liquid chromatography coupled to tandem mass spectrometry (UHPLC–MS/MS). Water deficit drastically reduced shoot growth, while it did not affect fruit production. In contrast, fruit production was enhanced by mycorrhization regardless of the water treatment. The main tissue affected by water stress was the root system, where huge rearrangements in different nutrients and stress-related and growth hormones took place. Abscisic acid content increased in every tissue and fruit developmental stage, suggesting a systemic response to drought. On the other hand, jasmonate and cytokinin levels were generally reduced upon water stress, although this response was dependent on the tissue and the hormonal form. Finally, mycorrhization improved plant nutritional status content of certain macro and microelements, specially at the roots and ripe fruits, while it affected jasmonate response in the roots. Altogether, our results suggest a complex response to drought that consists in systemic and local combined hormonal and nutrient responses.

**Supplementary Information:**

The online version contains supplementary material available at 10.1007/s00425-023-04192-2.

## Introduction

Plants are constantly submitted to various environmental abiotic stresses that substantially determine their growth and physiological status. Among all the abiotic stresses, drought can be considered as one of the most detrimental for plant performance, and it is expected to be more ubiquitous and recurrent due to climate change. In addition, its combination with other (a)biotic stresses may lead to significant losses in agricultural production (Challinor et al. 2014; reviewed by Ahluwalia et al. 2021). Conversely, there are reports showing that a mild and controlled water stress may successfully improve fruit quality in different crops such as tomato (*Solanum lycopersicum*; Guida et al. 2017; Wu et al. 2022). As sessile organisms, plants have developed different adaptive responses to cope with water stress, which go from morphological adaptations to complete rearrangements of the hormonal profile and transcriptome in above- and below-ground tissues (Tardieu et al. 2018). The root system is of major importance in plant acclimation to water stress, as its response may eventually have a great impact on the rest of the plant. For instance, upon drought, root system architecture is reshaped to optimize water absorption, while the accumulation of compatible osmolytes, like proline, reduces the root water potential, thus improving water uptake from the dry soil (Tardieu et al. 2018; Gupta et al. 2020). Despite displaying effective strategies against drought, the physiological status of stressed plants is eventually poor, leading to stunting and reduced growth as well as to a severe drop in fruit production.

From the different players involved in plant acclimation to water stress, phytohormones could be considered as key modulators. Among all, abscisic acid (ABA) is considered one of the main regulators, as it is involved in controlling stomatal closure and root growth in front of drought (reviewed by Kishor et al. 2022). In addition, ABA may act in combination with other hormones from the jasmonate family, like the precursor 12-*oxo*-phytodienoic acid (OPDA), to strengthen stomatal regulation in front of water stress; or with free jasmonic acid (JA) and its conjugated active form, jasmonoyl isoleucine (JA-Ile), to display effective defense mechanisms to other stresses like herbivory (Savchenko et al. 2014; Nguyen et al. 2016). Jasmonates are also essential in regulating plant interactions with beneficial microorganisms, and together with salicylic acid (SA), they constitute the backbone of the plant defense system against microbial pathogens (Pieterse et al. 2012; Betsuyaku et al. 2018). One of the main impacts of drought are deep alterations of plant growth and development, which are additionally regulated by another set of hormones: auxins and cytokinins. Shoot tip-synthesized indole-3-acetic acid (IAA), the main active form of auxin, is responsible for the establishment of the apical dominance, root formation and cell expansion, and, therefore, growth promotion (reviewed by Du et al. 2020). On the other hand, cytokinins enhance cell division, they play a key role in nutrient allocation, senescence, and they significantly influence fruit set and development (Matsuo et al. 2012; Wu et al. 2021). In addition, the occurrence of different forms of active cytokinins, like 2-isopentenyl adenine (2iP) (and its precursor, isopentenyl adenosine [iPA]) and *trans*-zeatin (*t*-Z) (and its precursor, *trans-*zeatin riboside [*t-*ZR]) may vary depending on the plant organ (Kudoyarova et al. 2007; Hirose et al. 2008). Altogether, plant acclimation to water stress implies a complex regulation of this phytohormone network, which eventually has profound systemic and local physiological effects on the plant.

Despite the systemic implications of plant response to water stress, the regulatory function of the root system in this response should be especially considered. Apart from their role of providing the plant with water, roots are necessary to maintain proper nutrient contents in the plants and are essential to carry out effective defensive strategies against (a)biotic stress. For example, stomatal regulation by ABA is dependent on the fluxes of potassium ions (K^+^) in the guard cells, which is directly provided by the roots, and cytokinin function, and therefore growth promotion, is tightly linked to nitrogen (N) and phosphorus (P) acquisition by the root (Hirose et al. 2008; Lawson and Matthews 2020). In addition, roots modulate hormone levels, like cytokinins and ABA, in distant plant organs. In the case of cytokinins, for example, *t*-ZR is synthesized in the roots and transported through the xylem vessels to the target tissue, where it is converted to its active form, *t*-Z (Matsumoto-Kitano et al. 2008). Peptide signals coming from the root of *Arabidopsis thaliana* are essential to trigger leaf ABA accumulation in front of drought, and direct ABA transport from the root influences leaf concentrations of that hormone under salinity (Li et al. 2018; Takahashi et al. 2018). Moreover, water deficit reduces nutrient absorption of most macro- and micronutrients through the roots, either by reducing their mobility in the soil (e.g., N or P) or modifying their availability (e.g., Fe) (reviewed by Ahanger et al 2016). For all the previous reasons, roots are essential modulators of plant development and systemic response to water stress.

The root system is also the main playground of plant interactions with beneficial microorganisms that may aid the plant to better cope with water scarcity (Koskey et al. 2021). One of the most abundant are arbuscular mycorrhizal fungi (AMF), which can establish beneficial interactions with the roots of up to the 80% of terrestrial plants. In this symbiosis, plants benefit from the increased water and nutrient availability given by the AMF in exchange of photoassimilates (Parniske 2008). Upon drought, AMF presence generally improves the plant water status due to the high capacity and efficiency of the fungal extraradical hyphae to absorb water. Besides, mycorrhizal colonization enhances macro- and micronutrient uptake, especially that of P and Cu (Lehmann and Rillig 2015; Ahanger et al 2016). In addition, this root–microbe interaction has a great impact in the root system response to water stress, like improving specific antioxidant and enzymatic activity, varying osmolyte accumulation, and modifying root growth patterns (Bárzana et al. 2015; Huang et al. 2017; Abdalla and Ahmed 2021). Besides, AMF also induce resistance to necrotrophic pathogens, like *Botrytis cinerea*, in tomato leaves (Sanmartín et al. 2020). Consequently, AMF have arisen as a promising sustainable solution to improve agricultural production in front of stressful situations like water deficit (Zhang et al. 2019; Biel et al. 2021).

Even though roots are essential to modulate plant–microbe interactions and water stress responses, they have been overlooked players in hormonal regulation of plant acclimation to water stress. However, the recent studies have shown that water stress-induced hormonal changes in roots influence shoot hormonal responses. De Ollas et al. (2018) suggested that JA export from roots could affect stomatal regulation in front of water stress through its interplay with leaf OPDA. Furthermore, mycorrhization induced a root-to-shoot signaling mediated by OPDA in front of water stress in white clover (*Trifolium repens*) (Fresno et al. 2023). Although these studies shed light on the differential hormonal responses between roots and leaves upon water stress, as well as the hormonal signaling between both tissues, other organs like fruits have remained unexplored. To evaluate whether or not fruits have specific hormonal responses in front of water stress, and to clarify to which extent they are related to root and leaf hormonal variations, we submitted tomato (*S. lycopersicum* cv. Moneymaker) plants, an economically relevant fruit-bearing species, to a mild water stress in the presence or absence of the AMF *Rhizoglomus irregulare*. In this work, we evaluated endogenous hormonal and nutritional contents in roots, leaves, and fruits at different developmental stages, as well as fruit production and quality parameters, throughout the water stress period. This work showed the root as a key hormonal coordinator in the plant acclimation response to water stress, having systemic implications in distant organs.

## Materials and methods

### Plant and fungal material

Surface-disinfected tomato (*S. lycopersicum* cv. Moneymaker) seeds were sown on multi-pot trays containing sterile perlite, watered with a ½ Hoagland solution and cultivated in a climatic chamber (Ibercex S.L., Spain; lamps: Philips Master TL-D 36W/840, Philips, The Netherlands) at 22 °C, with a 16:8 h (day:night) photoperiod and a photosynthetically active radiation (PAR) of 120 µmol m^−2^ s^−1^. Forty days after sowing, homogeneous tomato plants with two-to-three true leaves were root-cleaned and transplanted into 4 L pots filled with sterile sand and containing 10 g of a mycorrhizal bulk inoculum of the arbuscular mycorrhizal fungus *Rhizoglomus irregulare* (Blaszk, Wubet, Renker and Buscot; Sieverding, Silva and Oehl comb. nov.), isolated from a citrus nursery (Estaun et al. 1994). This inoculum was provided by the Institute of Agrifood Research and Technology (Barcelona, Spain). Non-mycorrhizal pots contained the same amount of an autoclaved inoculum. A microbial 20 μm filtrate of the original inoculum was added to non-mycorrhizal pots to homogenize microbial populations.

Plants were put inside a glass greenhouse on May 28th, 2022. To enhance mycorrhizal colonization, they were manually watered in a daily basis to full soil capacity with distilled water for 7 days. Thereafter, water irrigation was replaced with a ½ Hoagland solution with a 10% of the standard P concentration (0.1 mM) for 14 days. Finally, manual irrigation was substituted by an automated drop irrigation system that distributed a ½ Hoagland solution with a 33% of the standard P concentration twice a day, delivering 500 mL per plant every day. A Dosatron injector (Dosatron International LLC., Clearwater, FL, USA) was used to pump a concentrated stock nutrient solution into a deionized water line to finally obtain the desired nutrient concentration. Final pH of the solution was regularly monitored and kept at pH 6 all throughout the experiment. Cultivation inside the greenhouse ended August 10th, 2022. All the experiments were carried out at Servei de Camps Experimentals of the University of Barcelona, Barcelona (NE Spain).

### Irrigation treatments, sampling, and sample preparation

At 61 days after sowing (21 days after inoculation), mycorrhizal colonization was visually checked under a dissecting microscope. Afterward, half of the plants were submitted to a mild water stress treatment that consisted of a reduction in irrigation frequency. Water-stressed plants received one out of three drop irrigation waterings in comparison to well-watered plants, which resulted in 65% drop in water supply in comparison to control individuals. A more detailed explanation of the water stress treatment is shown in Suppl. Fig. S1. Samplings were performed every 7 days before dawn (3 a.m. local time), from the beginning of drought application (0 days after drought [dad]) to the point of maximum stress (14 dad).

In each sampling, maximal photochemical efficiency of photosystem II (*F*_v_/*F*_m_) was determined with a Mini PAM II yield analyzer (Heinz Walz GmbH, Effeltrich, Germany) at one fully developed and non-senescent leaflet for each plant. The same leaflet was used to establish the leaf water status. Afterward, all the tomato fruits from each plant were collected, weighed, and classified into three developmental stages based on Skolik et al. (2019). Due to the high diversity of stages in each plant, three main fruit developmental stages were defined: underdeveloped stage (from DS04 to mature green), intermediate stage (from breaker to pink), and ripe stage (light red and red ripe). Total production per plant was calculated, and tomato diameter was measured. Additionally, three ripe tomatoes from each experimental condition were kept to further test fruit resistance to a postharvest decay by *Botrytis cinerea*. Finally, the whole plant was uprooted, and shoot and root fresh weights were quantified. A subset of roots was taken to determine mycorrhizal colonization using the gridline-intersect method after root staining with ink and vinegar (Suppl. Fig. S2; Giovannetti and Mosse 1980; Vierheilig et al. 1998). Finally, at the point of maximum water stress (14 dad), a pool of three leaves per plant, a subset of the root system, and a pool of fruits at the three previously described developmental stages were frozen in liquid nitrogen and kept at – 80 °C for further hormone and nutrient analyses. Five plants per experimental condition (*n* = 5) were used in root, shoot, and total fruit biomass assessment, as well as for root and leaf nutrient and hormonal analyses. Fruit hormone and nutrient analyses were performed using fruits from three plants per experimental condition (*n* = 3).

Due to the different characteristics and requirements of the different organs, root, leaf, and fruit samples were prepared for further nutrient and hormone analyses as follows. In the case of roots, a subset of the root system was freeze-dried and ground using a mixer mill (MM400, Verder Scientific, Haan, Germany) until a fine powder was obtained. This powder was subsequently used both for nutrient and hormone quantification. In the case of leaves, a pool of three frozen leaves per plant was used. On the one hand, a subset of this leaf pool was dried in a stove at 70 °C to constant weight and ground until obtaining a fine powder, which was used later for nutrient analyses. Another subset of the frozen leaf pool was ground in the mixer mill using liquid nitrogen to avoid sample defrosting and directly used for hormone quantification. Finally, in the case of fruits, a pool of frozen fruits from each developmental stage per plant was ground using a mixer mill in the presence of liquid nitrogen to avoid sample defrosting. A subset of this frozen fruit powder was used to determine fruit water content, another one was used for hormonal analysis, and the remaining frozen fruit powder was freeze-dried to perform nutrient analyses.

### Water and nutrient status

To calculate the plant water status, a fully developed non-senescent leaflet from each plant was selected, and its fresh (FW), turgid (TW), and dry (DW) weights were calculated. Relative water content (RWC) and hydration were calculated using the following formulas:

$${\text{RWC}} = 100 \times \left( {{\text{FW}} - {\text{DW}}} \right)/\left( {{\text{TW}} - {\text{DW}}} \right),$$

$${\text{Hydration}} = \left( {{\text{FW}} - {\text{DW}}} \right)/{\text{DW}}.$$

Soil water content (SWC) was also measured (Suppl. Fig. S3). At every sampling point, a homogeneous sample of fresh sand was taken, weighed (SFW) and dried inside a stove at 70 °C to constant weight, obtaining the soil dry weight (SDW). The soil of five pots per experimental condition (*n* = 5) were used. SWC was calculated using the following formula:

$${\text{SWC}} = 100 \times \left( {{\text{SFW}} - {\text{SDW}}} \right)/\left( {{\text{SDW}}} \right).$$

Frozen fruit powder was used to calculate fruit water content, following the same proceeding and formula as in the SWC calculation.

To determine carbon (C) and nitrogen (N) proportion in leaves, roots, and fruits at different developmental stages, 3 mg of a dry fine powder from each organ were weighed and put inside tin capsules. Biological samples were then analyzed by an organic elemental analyzer (Thermo EA-1112, Thermo Scientific, Milan, Italy) applying the standard conditions recommended by the supplier (combustion reactor at 1000 °C, reduction reactor at 680 °C, reactor chromatographic column at 45 °C, and helium and oxygen flows at 120 and 100 mL/min). Endogenous levels of phosphorus (P), sulfur (S), potassium (K), sodium (Na), calcium (Ca), magnesium (Mg), copper (Cu), iron (Fe), manganese (Mn), and zinc (Zn) were determined by inductively coupled plasma-optical emission spectroscopy (ICP-OES Optima 8300 analyzer, Perkin Elmer Inc., Waltham, MA, USA) after acid digestion of 50 mg (root and leaves) and 100 mg (fruits) of dry powder.

### Leaf, root, and fruit hormone quantification

Endogenous ABA, jasmonates (OPDA, JA and JA-Ile), SA, cytokinins (iPA, 2iP, *t*-ZR, and *t*-Z), and auxin (IAA) were extracted and quantified by ultrahigh-performance liquid chromatography coupled to tandem mass spectrometry (UHPLC-MS/MS). Ground fresh fruits (100 mg) at three different developmental stages, ground fresh leaves (100 mg) and freeze-dried roots (30 mg) were extracted with 250 μL of a methanol:2-propanol:glacial acetic acid (50:49:1, by vol.) mix. Deuterium-labeled standards (d6-ABA, d5-JA, d4-SA, d6-iPA, d6-2iP, d5-*t*-ZR, d5-*t*-Z, and d5-IAA) were added at the beginning of the process to estimate recoveries. Extracts were submitted to ultrasonication (Branson 2510 ultrasonic cleaner, Emerson Electric Co., Sant Louis, MO, USA) and vortexing for 30 min, followed by a 10 min centrifugation at 15,000 g (PrismR, Labnet International Inc., Edison, NJ, USA). The supernatant was collected, the pellet was re-extracted, and both supernatants were finally mixed. Finally, the extract was filtered through a hydrophobic 0.22 μm filter (Phenomenex Inc., Torrance, CA, USA). Hormone levels were analyzed by UHPLC–ESI–MS/MS as described by Müller and Munné-Bosch (2011). The UHPLC was coupled to a triple quadrupole mass spectrometer (QTRAP 4000, AB Sciex, Concord, Ontario, Canada). An LUNA C18 column (Phenomenex Inc.; 1.6 µm, 100 × 2.1 mm) was used. Solvent A was water with 0.05% acetic acid and solvent B was acetonitrile with 0.05% acetic acid. Flow rate was set at 0.6 mL min^−1^. Quantification was made considering recovery rates for each sample using the deuterium-labeled internal standards. Calibration curves for each analyte were generated using MultiQuantTM 3.0.1 software.

### Tomato inoculation with *Botrytis cinerea*

Ripe tomato fruit resistance to a postharvest decay agent was measured as a fruit quality parameter. A virulent strain of *B. cinerea* isolated from strawberries and stored in a glycerol stock at – 80 °C was cultivated for 20 days in mixed vegetable medium plates as described by Fernández et al. (2014) to induce fungal sporulation. Fungal spores from each plate were collected using a buffer containing 0.5 mg mL^−1^ glucose and 0.5 mg mL^−1^ KH_2_PO_4_, and filtered through a Miracloth mesh with a 22–25 µm-wide pore size (Merck, Darmstadt, Germany). The final fungal inoculum consisted of this spore filtrate with an adjusted concentration of (10^5^ spores/mL). At the point of maximum stress (14 dad), three ripe fruits from every experimental condition were selected and surface disinfected using 70% ethanol. The fruits were placed in individual holders inside wet chambers, which consisted in a box containing sterile pieces of paper towel soaked in sterile distilled water. Two 5 mm-wide and 5 mm-deep holes were done in each fruit, and 10 µL of the fungal inoculum were put in each hole. The wet chambers were placed inside a plant growing chamber at 22 °C with a 12:12 h (day:night) and a PAR of 80 µmol m^2^ s^−1^. Disease severity was assessed by measuring the lesion diameter caused by the pathogen after the first symptoms were clearly visible (76 and 96 h after the inoculation).

### Statistical analyses

The general effects of the factors ‘Time’, ‘Water Stress’, and ‘Mycorrhization’ and their interactions on root, shoot, and fruit biomass, on tomato mean diameter and on leaf hydration and RWC were evaluated by performing a three-way ANOVA for independent samples with interactions. This analysis was also applied to disease severity caused by *B. cinerea* in ripe fruits. To test the general effect of the factors ‘Water Stress’ and ‘Mycorrhization’ and their interaction on nutrient and hormonal contents at 14 dad, a two-way ANOVA for independent samples with interaction was carried out specifically for each plant organ (roots, leaves, underdeveloped, intermediate, and ripe fruits). Finally, to test the effect of ‘Time’ and ‘Water Stress’ and their interaction in root mycorrhization, a two-way ANOVA for independent samples with interaction was carried out. Data were transformed whenever necessary to achieve homoscedasticity and normality of the residuals. Every figure shows the significance of each factor and interactions, being considered significant at a probability level of *P* < 0.05. Non-significant effects were marked as NS.

Spearman rank correlation tests between hormone and nutrient concentration were performed using a total of 76 observations (*n* = 76), 20 of which corresponded to root data, 20 to leaf data, 12 to underdeveloped fruits, 12 to intermediate fruits, and 12 to ripe fruits. Correlations were considered significant at a probability level of *P* < 0.001. Statistical analyses were performed using RStudio (RStudio Team 2020).

## Results

### Tomato plant growth and water status, but not fruit production, were negatively affected by a mild water stress

**Fig. 1:**
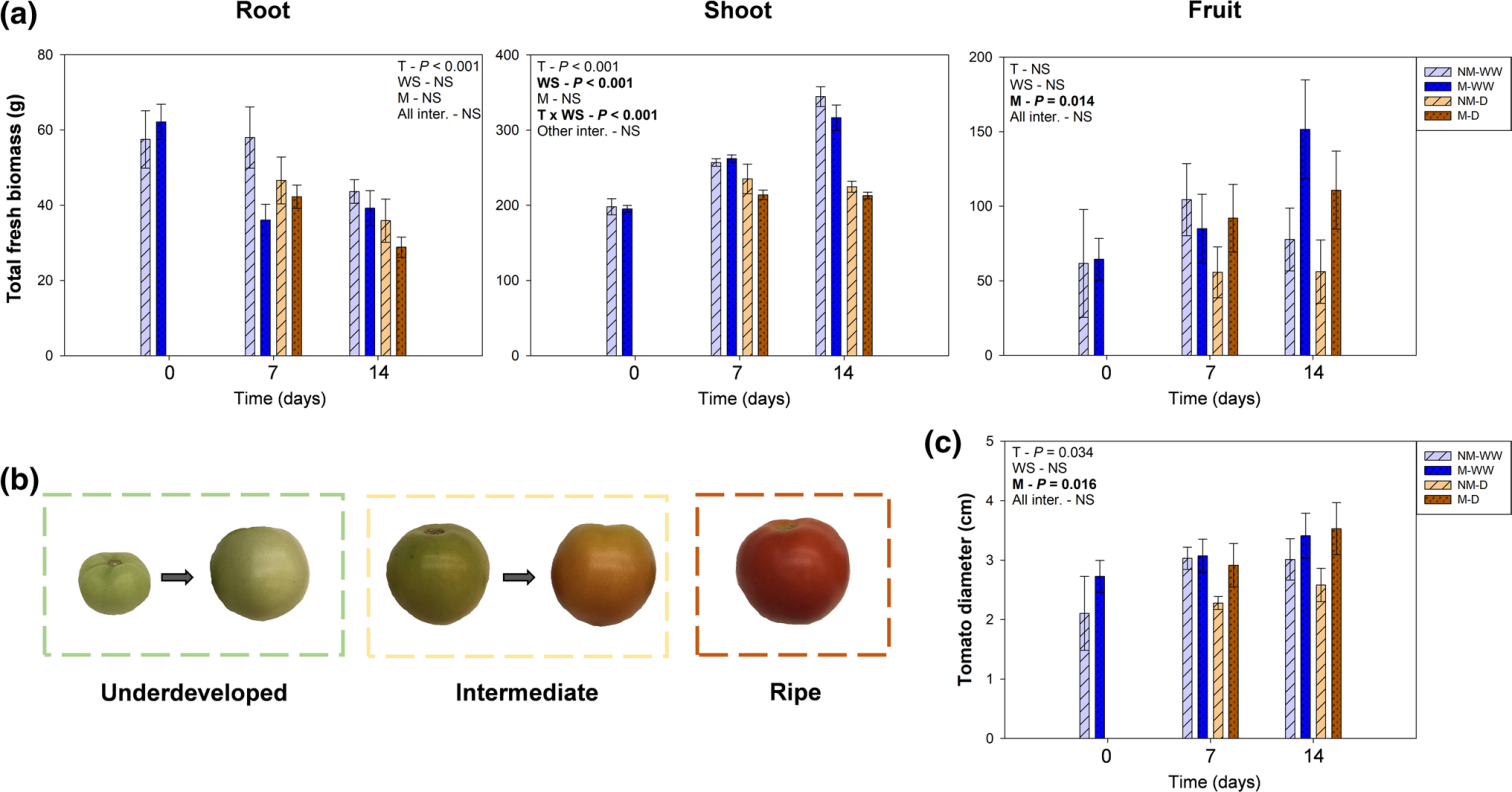
Water stress affects plant growth but not fruit production in a 14 day-long mild water stress period. **a** Root, shoot, and total fruit biomass of tomato plants inoculated (M) or non-inoculated (NM) with the arbuscular mycorrhizal fungus *Rhizoglomus irregulare* under well-watered (WW) or mild water stress (D) conditions. **b** Tomato fruits were separated into three main groups: underdeveloped, intermediate, and ripe. **c** Mean tomato diameter per plant. Data are means ± SE of *n* = 5 plants. Effects of ‘Time’ (*T*), ‘Water Stress’ (WS), ‘Mycorrhization’ (M), and all the interactions were evaluated by performing a three-way ANOVA

**Fig. 2:**
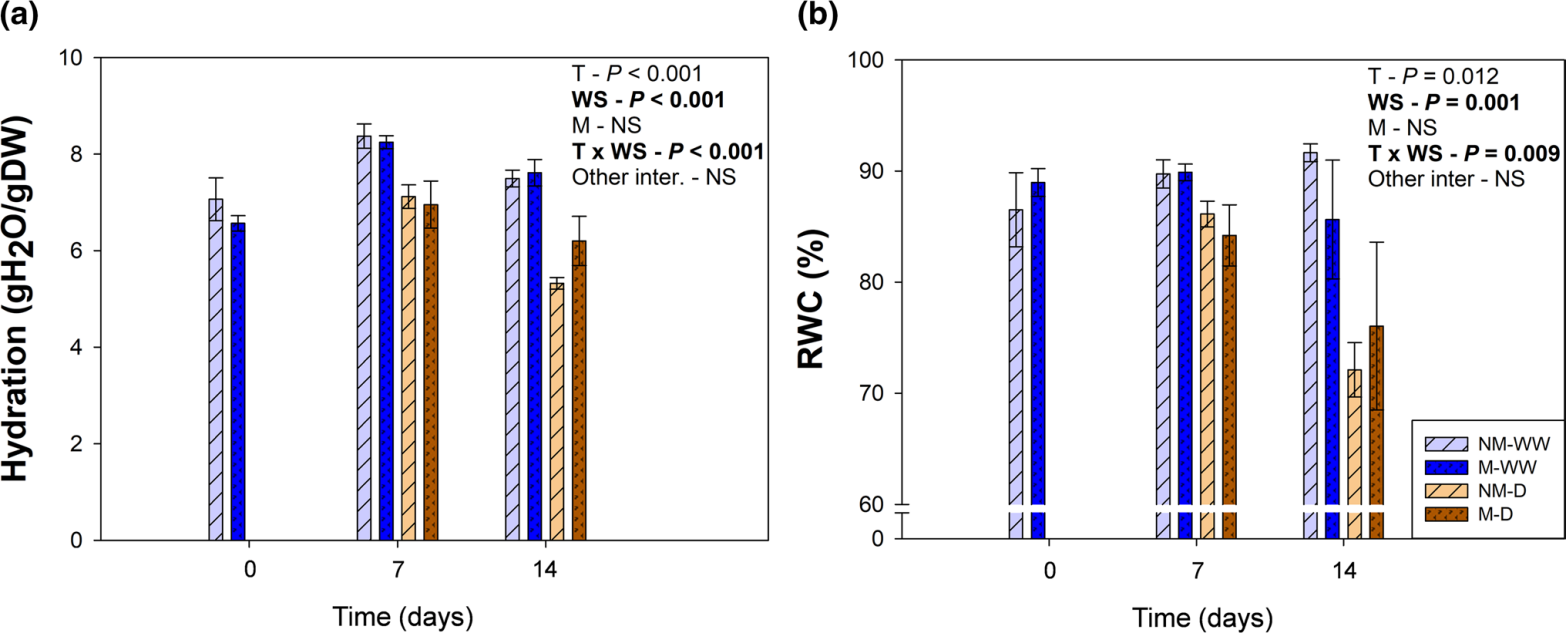
A mild water stress affects shoot water status. Hydration (**a**) and leaf relative water content (RWC) (**b**) of tomato plants inoculated (M) or non-inoculated (NM) with the arbuscular mycorrhizal fungus *Rhizoglomus irregulare* under well-watered (WW) or mild water stress (D) are represented. Data are means ± SE of *n* = 5 plants. Effects of ‘Time’ (*T*), ‘Water Stress’ (WS), ‘Mycorrhization’ (M), and all the interactions were evaluated by performing a three-way ANOVA

A 14 day-long mild water stress affected vegetative growth in an organ-specific manner. Despite water stress did not affect root growth, shoot growth over time was significantly stopped by water stress. Specifically, stressed plants showed a significant decrease in fresh shoot biomass of up to 40% at 14 dad in comparison to well-watered individuals (Fig. 1a). Conversely, total fruit biomass was unaltered by water stress, while mycorrhization significantly enhanced fruit production (especially at 14 dad) independently on the irrigation treatment (Fig. 1a). Fruit quality parameters like tomato diameter and fruit resistance to the postharvest decay fungus *B. cinerea*, were also quantified. Mycorrhization improved fruit diameter, although it had no effect on tomato resistance to *B. cinerea*. Surprisingly, fruits coming from water-stressed plants presented smaller lesion diameters than those from well-watered plants (Fig. 1c, Suppl. Fig. S4a). Water status of the shoot was negatively affected by water stress, with a significant reduction in leaf hydration and RWC (Fig. 2). However, this deterioration in water status did not lead to photoinhibition, with *F*_v_/*F*_m_ values above 0.80 throughout the experiment (Suppl. Fig. S5). Mycorrhization did not have any positive effect on leaf hydration or RWC, but mildly improved *F*_v_/*F*_m_. Overall, water deprivation led to a reduction in shoot but not in root growth. In addition, *R. irregulare* increased tomato fruit production and diameter without improving the plant water status.

### Water stress and mycorrhization triggered differential nutritional and hormonal responses in different plant tissues and fruit developmental stages

**Fig. 3:**
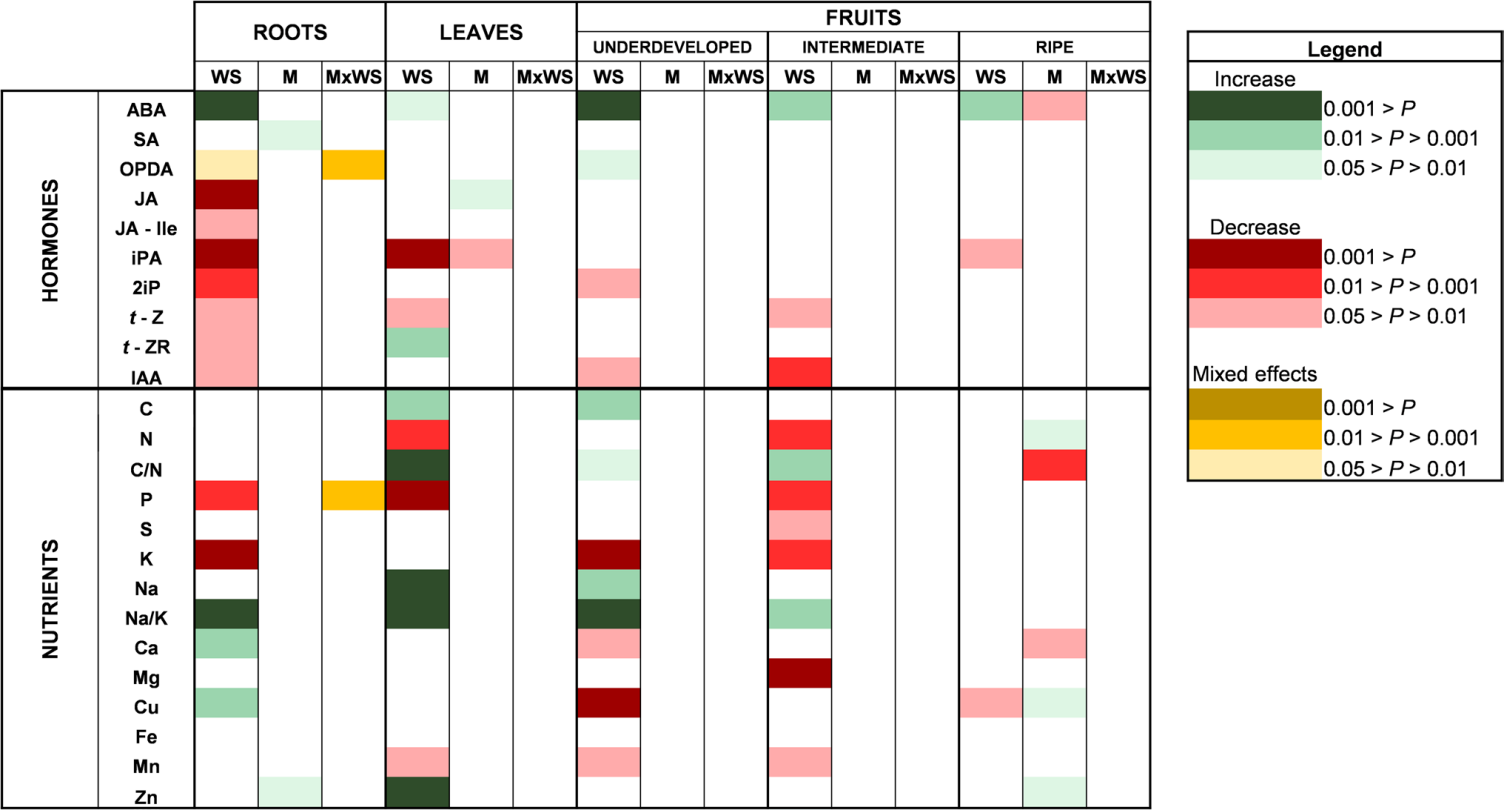
Water stress and mycorrhization induce profound changes in the hormonal and nutritional status of different plant organs. Positive and negative effects of the factor ‘Water Stress’ (WS), ‘Mycorrhization’ (M), and their interaction (M × WS) on each hormonal and nutritional parameter are represented in green and red colors, respectively. Those parameters that reacted in an opposite way in well-watered and water-stressed plants were described as mixed effects (yellow colors). Treatment effects were evaluated by performing a two-way ANOVA for each tissue, and color intensity refers to the significance of that effect based on the *P* value. Data correspond to samples taken 14 days after the beginning of the mild water stress treatment. *ABA* abscisic acid, *IAA* indole-3-acetic acid, *2iP* 2-isopentenyl adenine, *iPA* isopentenyl adenosine, *JA* jasmonic acid, *JA-Ile* jasmonoyl isoleucine, *SA* salicylic acid, *OPDA* 12-*oxo*-phytodienoic acid, *t-ZR* *trans*-zeatine riboside, *t-Z* *trans*-zeatin

A systemic shift in nutrient and hormonal contents took place upon water stress and mycorrhization, with organ-specific variations. All the analyzed parameters were affected either by the factor ‘Water Stress’, ‘Mycorrhization’ or their interaction at 14 dad to a greater or lesser extent, except for Fe, which remained unaltered in every plant organ. Water stress was the most important factor, affecting every tissue for most of the parameters. Mycorrhization and its interaction with water stress generated some specific responses in roots, leaves, and, especially, in ripe fruits (Fig. 3).

From a perspective of the phytohormone response to a mild water stress, the root system raised as a main regulatory hub, where nine out of ten analyzed hormones differentially accumulated or decreased. A systemic ABA increase was observed in every tissue or fruit developmental stage, which was partially alleviated by mycorrhization only in ripe fruits. The effect of water deprivation on other stress hormones such as jasmonates took place mainly on the root system, where mycorrhization also modulated OPDA response. Finally, a broad and general decrease in growth hormones like cytokinins (iPA, 2iP, *t*-Z, and *t*-ZR) and auxins (IAA) was observed in almost every tissue, with no effect of ‘Mycorrhization’ (Fig. 3). The effects of water stress and mycorrhization on stress and growth hormones will be further explained in the following sections of this paper.

From a nutritional perspective, water-stressed plants had an impoverished nutrient status, displaying some organ-specific variations. Systemic P starvation was clear in fruits at an intermediate developmental stage, leaves, and roots, where *R. irregulare* partially alleviated this drop (Fig. 3; Suppl. Fig. S6). In addition, a general increase in the Na/K ratio was broadly observed in almost every organ, with varying levels of Na, K, or both depending on the organ (Fig. 3). Other nutrients had markedly organ-specific responses. For instance, C and N concentrations, and the C/N ratio, were not affected by water stress in roots. However, C and C/N ratio increased in leaves and fruits (underdeveloped and intermediate), while C/N ratio decreased in ripe fruits because of mycorrhization. On the contrary, N levels were reduced by water stress in leaves and intermediate fruits, and partially improved by mycorrhization in ripe tomatoes (Fig. 3, Suppl. Fig. S7). Besides, water stress had a positive effect on Cu in roots, although the opposite occurred in fruits, where mycorrhization alleviated this drop in ripe tomatoes (Fig. 3, Suppl. Fig. S8). Finally, water stress-derived Mn starvation took place in leaves and fruits (underdeveloped and intermediate), while Zn increased in leaves due to the effect of water stress, and in roots and ripe fruits in the presence of *R. irregulare* (Fig. 3; Suppl. Fig. S9). Other nutrients like Fe and S remained mostly unaffected (Suppl. Fig. S8, S10).

Overall, the hormonal and nutritional profile was clearly affected by water stress, generating both systemic and organ-specific responses depending on the analyzed parameter. To better comprehend the physiological processes underlying these responses, the effects of water stress and mycorrhization on certain hormones (stress and growth-related hormones) and nutrients (Na, K, Ca, and Mg), as well as the specific effects of mycorrhization by *R. irregulare*, will be explained in detail in the following sections of this paper.

### Water stress triggered a systemic ABA and a root-focused mycorrhiza-mediated jasmonate response

**Fig. 4:**
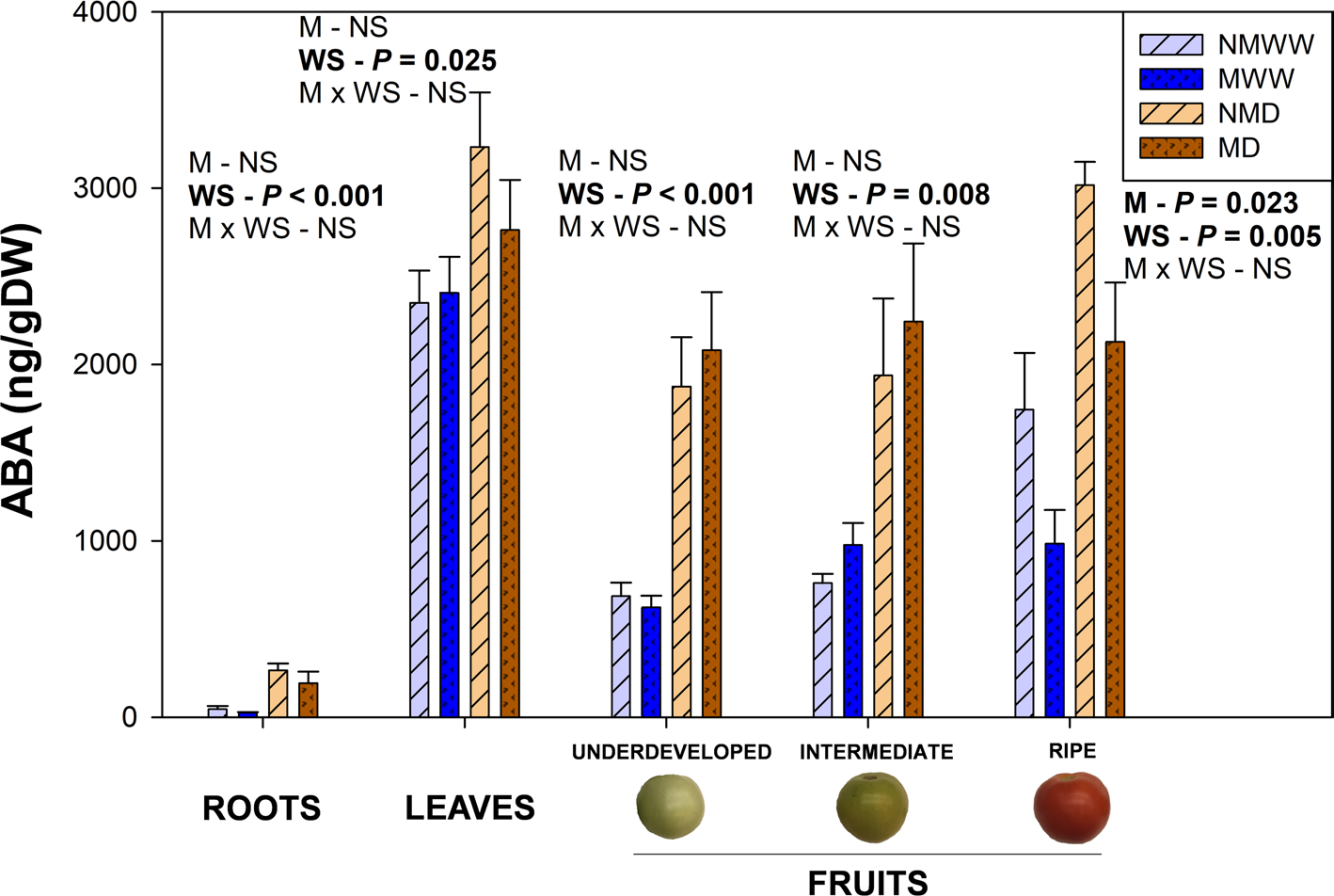
Water stress induces an abscisic acid (ABA) systemic response. Endogenous ABA concentration in roots, leaves, and fruits at different developmental stages of tomato plants inoculated (M) or non-inoculated (NM) with the arbuscular mycorrhizal fungus *Rhizoglomus irregulare* under well-watered (WW) or mild water stress (D) are represented. Data are means ± SE of *n* = 5 plants for roots and leaves, and of *n* = 3 for developmental fruit stages measured 14 days after the beginning of the mild water stress treatment. Effects of ‘Water Stress’ (WS), ‘Mycorrhization’ (M), and their interaction (M × WS) were evaluated by performing a two-way ANOVA for each tissue. Factors with a statistical significance (*P* < 0.05) are highlighted in bold letters or marked as NS when non-significant (*P* > 0.05)

Water stress induced a systemic increase in ABA endogenous concentration in roots, leaves, and fruits at every developmental stage 14 days after the beginning of the water stress treatment (Fig. 4). However, the intensity of this response was highly dependent on the organ and, in the case of fruits, on the developmental stage. For instance, roots, underdeveloped and intermediate fruits displayed the steeper increases, followed by ripe fruits and, finally, leaves. In addition, the root system was the organ with the lowest ABA concentration in comparison to leaves and fruits. Finally, the factor ‘Mycorrhization’ significantly and exclusively attenuated this response in ripe fruits.

**Fig. 5:**
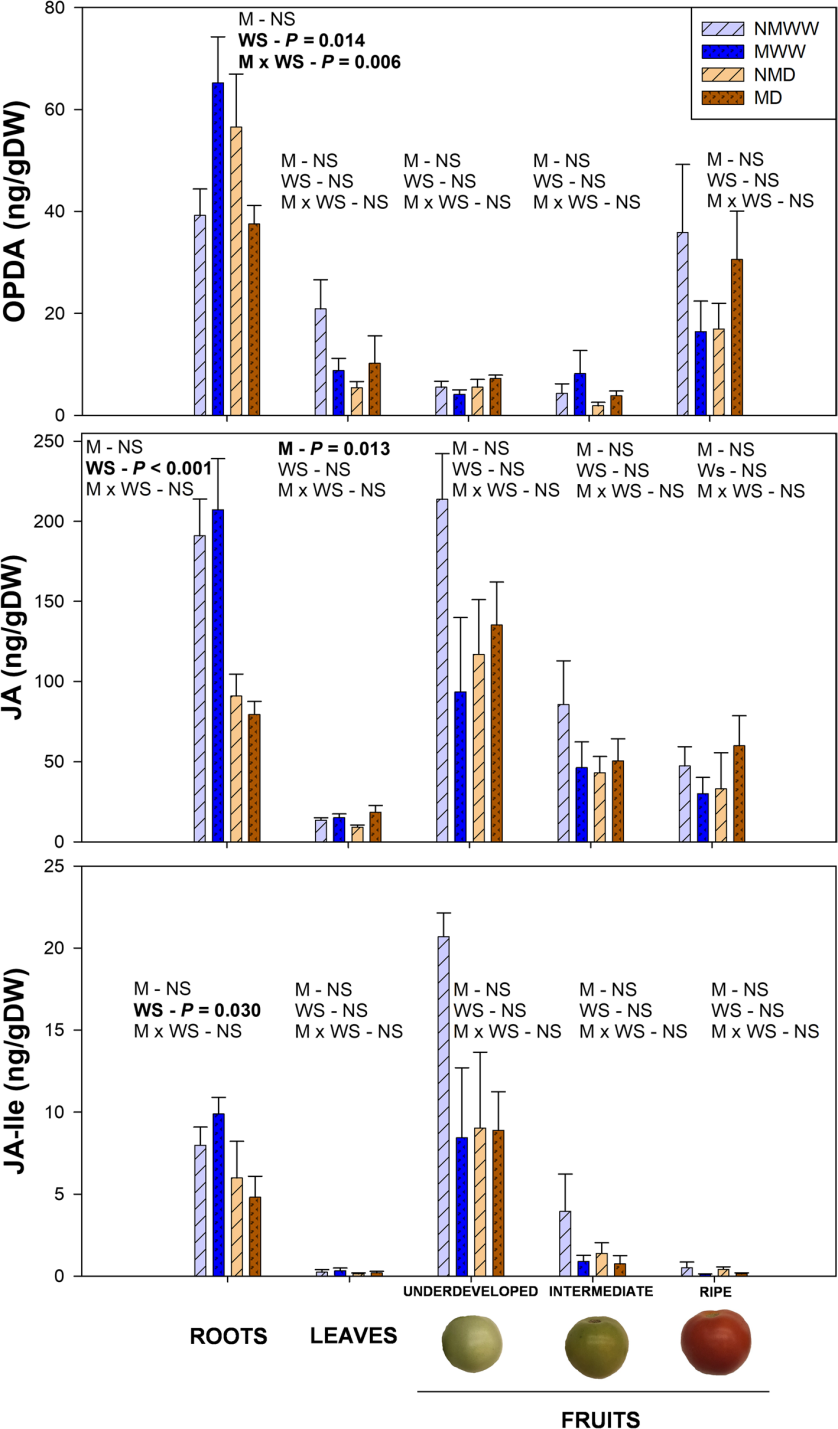
Jasmonate contents are affected by water stress and mycorrhization at different plant organs. Endogenous levels of the jasmonates precursor 12-*oxo*-phytodienoic acid (OPDA), free jasmonic acid (JA) and its conjugated form, jasmonoyl isoleucine (JA-Ile) in roots, leaves, and different fruit developmental stages of well-watered (WW) and water-stressed (D) tomato plants inoculated (M) or non-inoculated (NM) with the arbuscular mycorrhizal fungus *Rhizoglomus irregulare*, are shown. Data are means ± SE of *n* = 5 plants for roots and leaves, and of *n* = 3 for developmental fruit stages 14 days after the beginning of the mild water stress treatment. Effects of ‘Water Stress’ (WS), ‘Mycorrhization’ (M), and their interaction (M × WS) were evaluated by performing a two-way ANOVA for each tissue. Factors with a statistical significance (*P* < 0.05) are highlighted in bold letters or marked as NS when non-significant (*P* > 0.05)

Unlike ABA, the effect of ‘Water Stress’ on jasmonate endogenous levels was limited to roots, with a significant reduction in free JA and JA-Ile, and a mycorrhiza-dependent response for the precursor OPDA (with a significant interaction). In well-watered plants, root OPDA was significantly higher in mycorrhizal plants individuals, and when water stress was applied, OPDA concentration was reduced only in the presence of *R. irregulare* (Fig. 5). The influence of the AMF was extended to leaves, where mycorrhization significantly increased free JA, especially in stressed plants, but not of the precursor nor of the active conjugated form. In addition, fruit jasmonates were completely unaffected neither by water stress nor by mycorrhization. Finally, the concentration of each jasmonate form was highly organ dependent. For instance, roots were rich in OPDA and free JA, while JA-Ile content was lower in comparison to other tissues. Leaves displayed relatively lower contents of every jasmonate, while fruits tended to accumulate more OPDA in the ripe stage and JA and JA-Ile in underdeveloped and intermediate stages (Fig. 5).

### Cytokinin response to water stress is highly dependent on the plant organ and cytokinin type

**Fig. 6:**
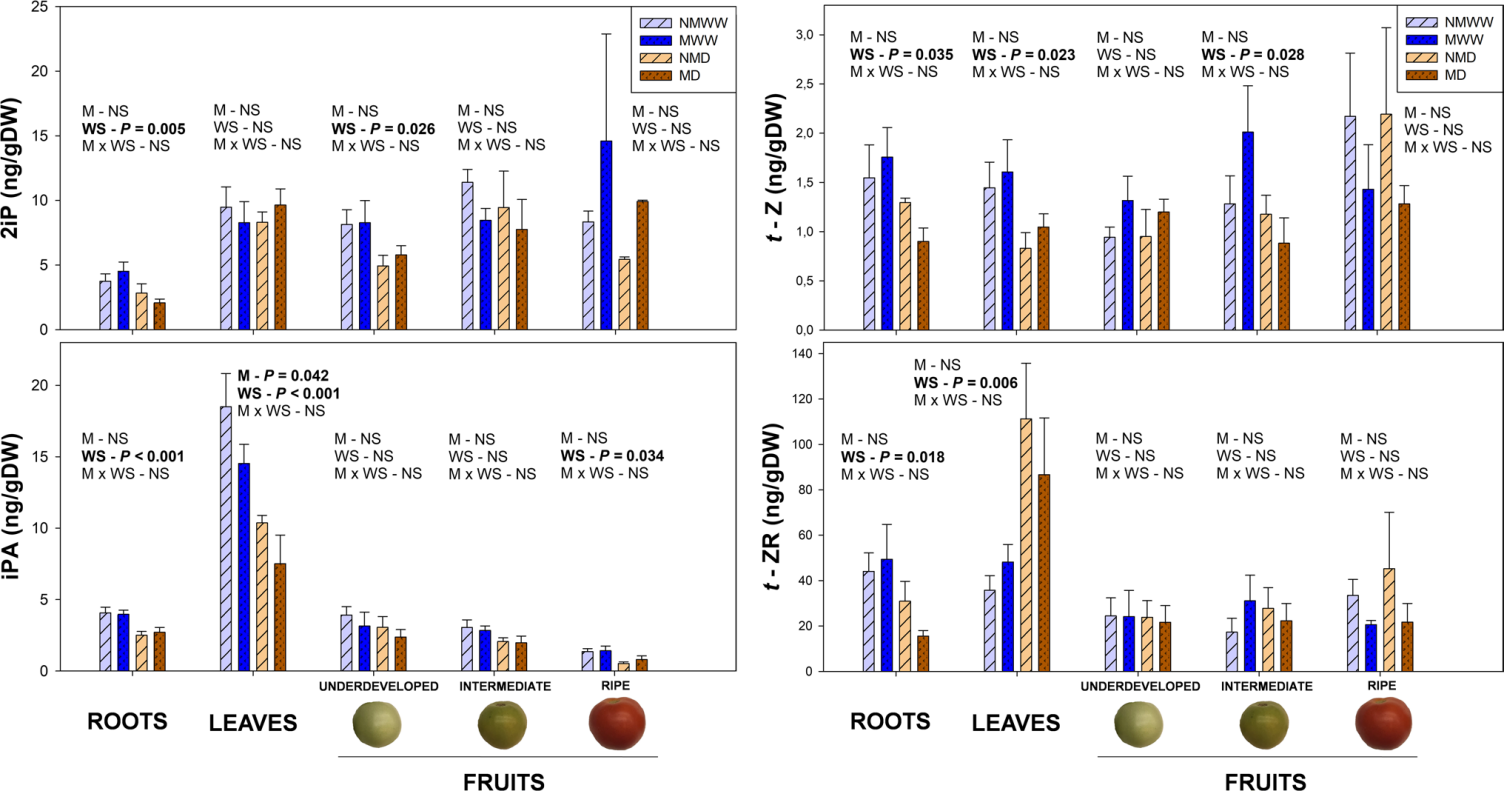
A mild water stress affects endogenous contents of various cytokinin forms in different plant organs. Endogenous levels of the active cytokinins 2-isopentenyl adenine (2iP) and *trans*-zeatin (*t*-Z), and their respective precursors, isopentenyl adenosine (iPA), and *trans*-zeatin riboside (*t*-ZR) in roots, leaves, and different fruit developmental stages of well-watered (WW) and water-stressed (D) tomato plants inoculated (M) or not (NM) with the arbuscular mycorrhizal fungus *Rhizoglomus irregulare*, are represented. Data are means ± SE of *n* = 5 plants for roots and leaves, and of *n* = 3 for developmental fruit stages 14 days after the beginning of the mild water stress treatment. Effects of ‘Water Stress’ (WS), ‘Mycorrhization’ (M), and their interaction (MxWS) were evaluated by performing a two-way ANOVA for each tissue. Factors with a statistical significance (*P* < 0.05) are highlighted in bold letters or marked as NS when non-significant (*P* > 0.05)

Water stress significantly reduced cytokinin endogenous concentration in a tissue and cytokinin-type dependent manner. Among all the different analyzed organs, the root system was the most affected by the factor ‘Water Stress’, with a consistent and significant reduction in both active forms and precursors of iP and *t*-Z-type cytokinins. This observation was not extended to other organs like leaves, which suffered differential responses depending on the cytokinin type. On the one hand, leaf *t*-Z levels were reduced by water deprivation, which was concurrent with an accumulation of the precursor *t*-ZR. Conversely, this pattern was not observed for 2iP, which remained unaltered by water stress, nor for the precursor iPA, which was significantly reduced and negatively influenced by mycorrhization. Regarding fruits, only a significant decrease in 2iP was detected in underdeveloped tomatoes, as well as a mild reduction of iPA in ripe fruits. *t*-Z type cytokinins in fruits were unaltered by any factor. Finally, it is noticeable that 2iP was the most abundant active cytokinin in every tissue and developmental stage, with approximately five-to-six times higher concentrations than those of *t*-Z (Fig. 6). A similar trend to that of cytokinins was observed for the auxin IAA. A mild negative effect of water stress was observed in roots, especially in non-mycorrhizal individuals, and underdeveloped fruits, but no effect on leaves was observed. In contrast to cytokinins, water stress induced a strong reduction in IAA levels in fruits at an intermediate developmental stage (Suppl. Fig. S11). Altogether, water stress severely influenced growth-related hormone levels, especially cytokinins, triggering differential responses highly dependent on the plant organ and cytokinin type, being the root system one of the most affected organs.

### Mild water stress induced a systemic shift in Na and K concentration with organ-specific dynamics

**Fig. 7:**
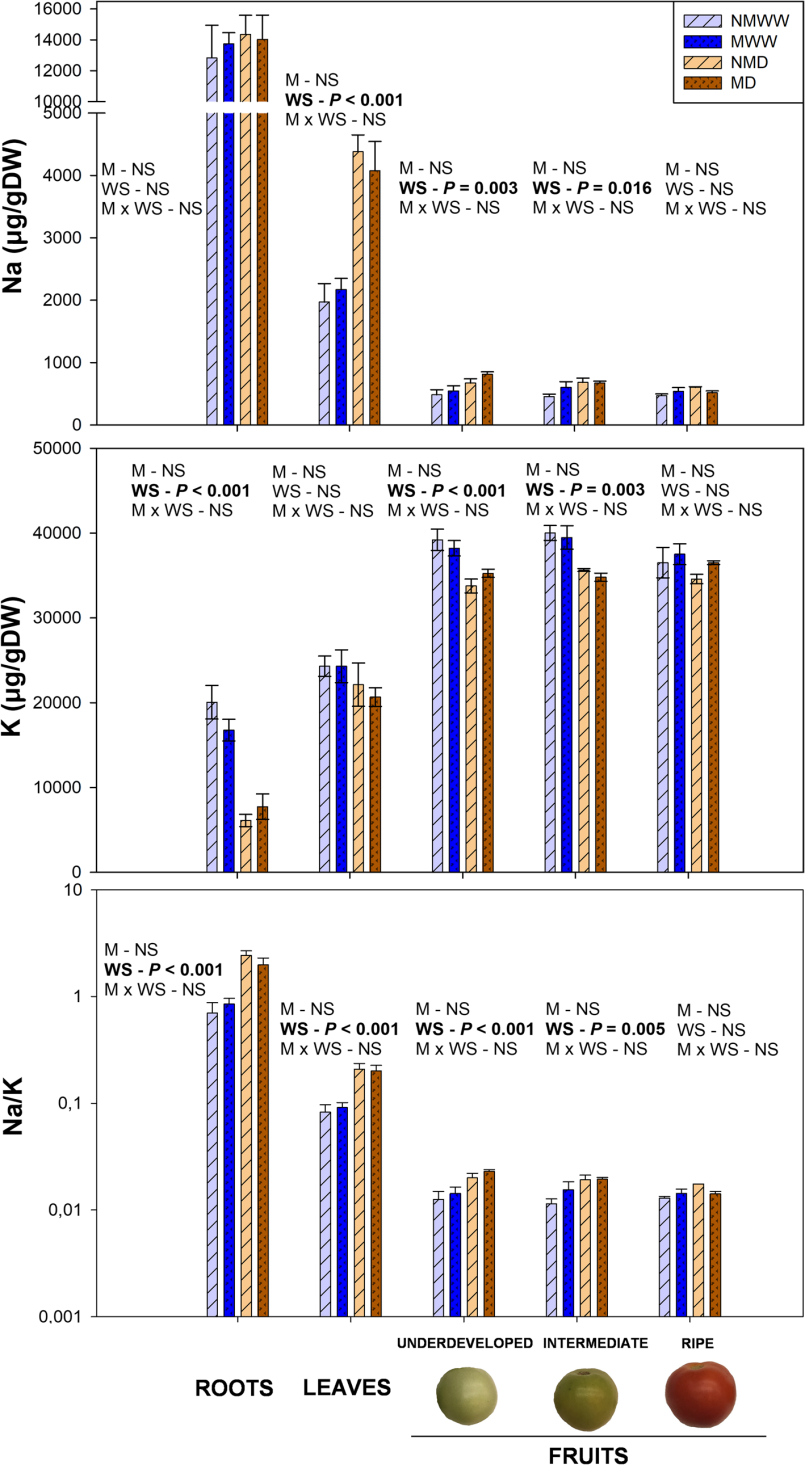
Mild water stress strongly influences sodium (Na), potassium (K) and the Na/K ratio in roots, leaves, and different fruit developmental stages. Endogenous levels of these elements in well-watered (WW) and water-stressed (D) tomato plants, inoculated (M) or non-inoculated (NM) with the arbuscular mycorrhizal fungus *Rhizoglomus irregulare*, are represented. Data are means ± SE of *n* = 5 plants for roots and leaves, and of *n* = 3 for developmental fruit stages 14 days after the beginning of the mild water stress treatment. Effects of ‘Water Stress’ (WS), ‘Mycorrhization’ (M), and their interaction (MxWS) were evaluated by performing a two-way ANOVA for each tissue. Factors with a statistical significance (*P* < 0.05) are highlighted in bold letters or marked as NS when non-significant (*P* > 0.05)

Water deprivation induced a marked readjustment in Na and K levels in different tissues at 14 dad, although the extent of this regulation was highly organ-dependent. In roots, K concentration was severely reduced by the factor ‘Water Stress’, while Na levels remained unaffected. As a result, Na/K ratio increased. In leaves, conversely, K content was unaltered by water stress, but Na concentration was sharply risen, leading as well to a higher Na/K ratio. Fruits at underdeveloped and intermediate developmental stages suffered subtle decreases and increases for both K and Na, respectively, while ripe fruits were completely unaffected by water stress (Figs. 3, 7). Thus, fruits were the only organs in which a dual regulation of both ions took place. Finally, it is noteworthy the abrupt differences in the concentration of both elements between organs, being the roots the organ with highest Na concentrations and fruits the ones with the highest K levels. Mycorrhization did not have a significant effect for any of the two elements in any organ or developmental stage (Fig. 7).

Other elements like Ca and Mg were as well affected by water stress. Ca behavior was different depending on the organ, with a significant increase in roots and a slight drop in underdeveloped fruits. Mycorrhization significantly reduced Ca content in ripe fruits. Leaves and fruits at intermediate developmental stage were unaffected. In the case of Mg, this element was only altered by water stress at intermediate fruits, and no other tissues were affected (Suppl. Fig. S12). In summary, water stress had significant implications in ion regulation, especially of the Na–K pair, with different intensities depending on the plant organ and fruit developmental stage.

### Mycorrhization improved the hormonal and nutritional status at different organs

**Fig. 8:**
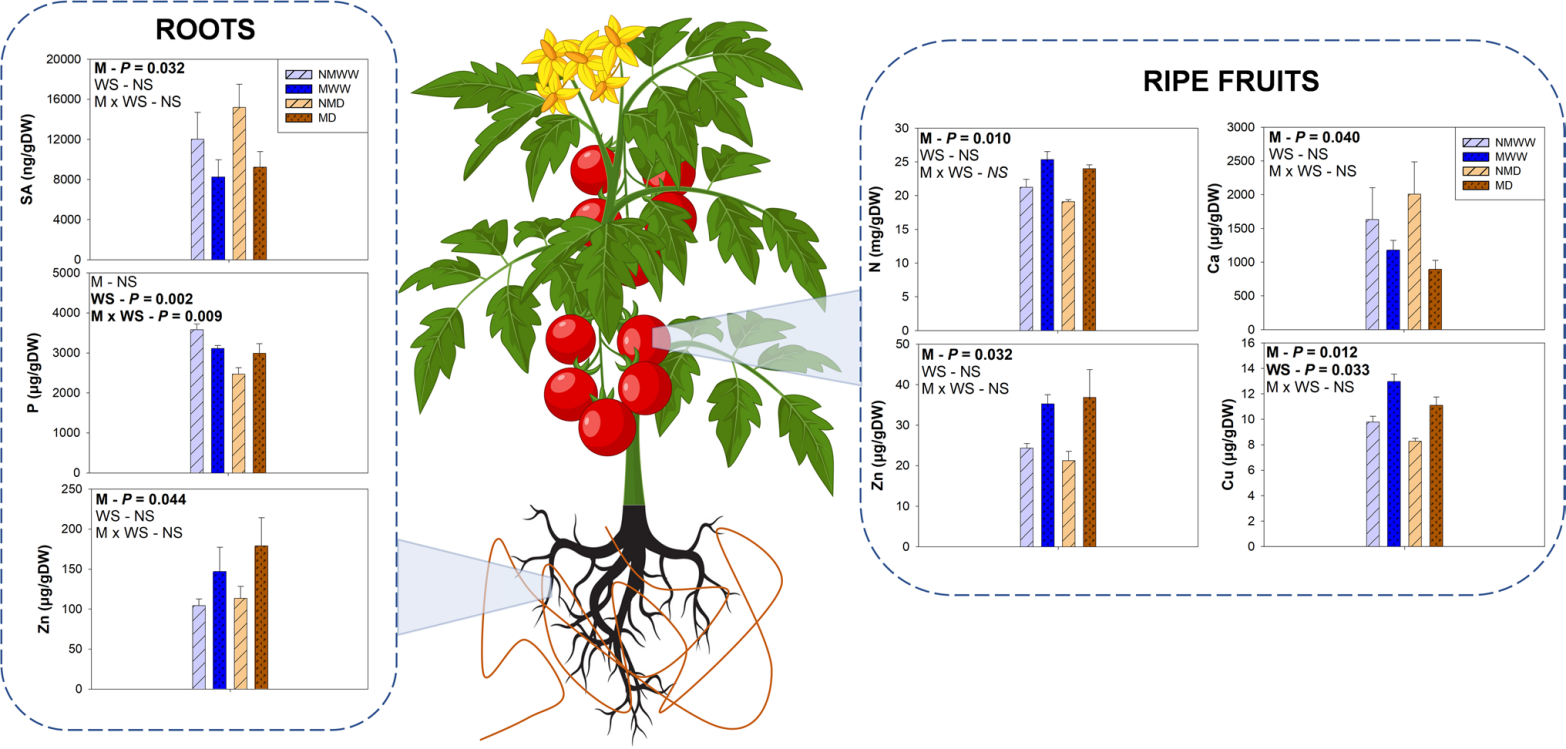
Mycorrhization with the arbuscular mycorrhizal fungus *Rhizoglomus irregulare* improves nutrient and hormonal status in roots (left panel) and ripe fruits (right panel). Endogenous content of nitrogen (N), phosphorus (P), calcium (Ca), copper (Cu), zinc (Zn), and salicylic acid (SA) 14 days after the beginning of a mild water stress treatment in mycorrhizal (M) and non-mycorrhizal (NM) well-watered (WW) and water-stressed (D) plants are shown. Data are means ± SE of *n* = 5 plants for roots and leaves, and of *n* = 3 for developmental fruit stages. Effects of ‘Water Stress’ (WS), ‘Mycorrhization’ (M), and their interaction (MxWS) were evaluated by performing a two-way ANOVA for each tissue. Factors with a statistical significance (*P* < 0.05) are highlighted in bold letters or marked as NS when non-significant (*P* > 0.05)

The presence of the AMF *R. irregulare* improved the nutritional status of roots and ripe fruits. To begin with, water stress-derived P starvation was significantly alleviated by the fungus, as well as Zn absorption, which was significantly enhanced. This improvement in Zn content was translated to ripe fruits, where mycorrhizal tomatoes displayed higher levels of this metal regardless of the water treatment. Even though Cu concentration in roots was not affected by water stress nor by mycorrhization, its levels were significantly increased in the ripe fruit by *R. irregulare* both in well-watered and stressed plants (Fig. 8). Finally, N was also enhanced in ripe fruits and, as explained before, Ca concentration was reduced in the presence of the AMF (Fig. 8; Suppl. Fig. S7, S12). The influence of mycorrhization was limited to ripe fruits, having no influence on previous tomato developmental stages. Regarding hormone status, besides the previously explained attenuation of the ABA response in ripe fruits (Fig. 4), or its influence in root and leaf jasmonate dynamics (Fig. 5), colonization by *R. irregulare* also enhanced SA levels in roots in both water treatments (Fig. 8, Suppl. Fig. S13). To sum up, the presence of *R. irregulare* improved the root nutritional and hormonal status, which was translated into a nutritional improvement in ripe fruits, but not in underdeveloped or intermediate tomatoes.

## Discussion

Water stress negatively impacts plant development by altering water relations, reducing nutrient uptake or impairing photosynthetic activity (Sun et al. 2020). As a result, plants have developed acclimation responses that may take place at specific tissues and/or extend systemically to the rest of the plant (Suzuki et al. 2013; Kollist et al. 2019). In this study, we monitored the acclimation responses of Moneymaker tomato plants to a mild water stress in the presence or absence of an AMF (*R. irregulare*), focusing on three specific plant organs: roots, leaves, and fruits. Our results showed that water stress led to a general nutritional impoverishment and a complete hormonal rearrangement in different tomato plant organs, being the roots the most affected one (9 out of 10 hormones affected), followed by leaves and underdeveloped fruits (4 out of 10) and intermediate and ripe fruits (3 and 2 out of 10, respectively). Even though some hormones (i.e., ABA) presented a systemic response, others like jasmonates or cytokinins showed tissue-specific variations upon water stress. Altogether, the root system stood as a hormonal regulatory hub in response to this stress, having profound hormonal changes that progressively faded in distant tissues like leaves and fruits. In addition, mycorrhization by *R. irregulare* improved fruit production and quality, as well as the nutritional and hormonal status, not only in the roots but in the rest of organs.

Even though water stress reduced shoot growth, leaf hydration, and leaf RWC, root growth and total tomato fruit biomass remained unaffected. These results agree with the previous studies which have reported that controlled reduction of irrigation (i.e., regulated deficit irrigation) has minor effects on tomato production, while it may improve several fruit quality parameters (Lu et al. 2019). Indeed, water stress slightly increased fruit resistance to the fungal pathogen *B. cinerea*, which could have potential application in improving fruit shelf life. The presence of AMF, however, did not ameliorate fruit resistance to the fungus, although the previous reports have shown that leaves of mycorrhizal plants are more resistant to the same pathogen (Sanmartín et al. 2020). Nevertheless, *R. irregulare* increased total fruit biomass and mean tomato diameter. This coincides with the previous literature, although all results taken together suggest that the extent of the beneficial effect of AMF in fruit production and quality may vary depending on the plant species and variety, growing conditions, and drought intensity (Subramanian et al. 2004; Zhang et al. 2019). Generally, AMF-mediated enhancement in fruit production relies on the improved water absorption conferred by the fungus (Abdalla and Ahmed 2021; Fresno et al. 2023), although in the present study, leaf water status remained unaffected by mycorrhization. Thus, AMF-mediated improvement of fruit biomass and size may be explained by the increase in the content of nutrients like N, Cu, and Zn, which are all essential to enhance fruit yield and quality in tomato plants (Adams et al. 1978; Cheng et al. 2021; Almendros et al. 2022; Lafuente et al. 2023).

Phytohormones modulate plant responses to (a)biotic stress. In front of water stress, ABA constitutes one of the main molecules regulating the plant defense responses. In our experiment, there was a systemic surge in ABA contents, although leaves and ripe fruits displayed milder increases. This variation in response intensity, as well as in endogenous ABA concentration between tissues might be explained by the different functions that this hormone has in each of them. ABA modulates root growth and architecture upon drought, while in leaves, it acts on the hydraulic regulation of stomatal guard cells (Kishor et al. 2022; Zhang et al. 2022a). In fruits, as well as in the shoot, it is involved in the regulation of ethylene synthesis, the main modulator of climacteric fruit ripening (Zhang et al. 2008; Dodd et al. 2009). Mycorrhization has been proven to improve ABA responses in leaves and roots in front of water stress (Chitarra et al. 2016). However, in this experiment, we proved that this improvement was extended to ripe fruits, suggesting that AMF may be able to modulate fruit ripening through the regulation of this hormone. Exploring this fact in future experiments would be of great interest due to its potential agricultural application.

**Fig. 9:**
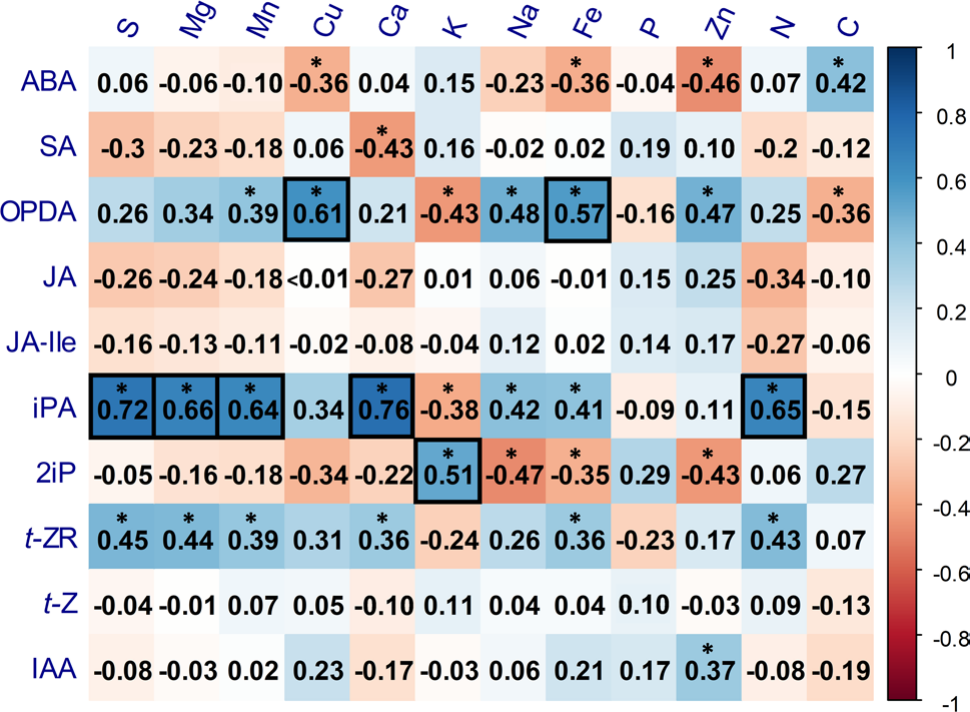
Spearman rank correlation matrix between hormone (left) and nutrient (top) contents. Correlations were made using data from roots, leaves, and fruits at different developmental stages of control and water-stressed tomato plants 14 days after the beginning of a mild water stress treatment (*n* = 76). Rho values for each correlation are displayed in each box. Correlations with a *P* value below 0.001 (*P* < 0.001) were considered as significant and marked with an asterisk. Strong (rho > 0.5) and significant correlations are highlighted with a black frame. The color gradient represents ranges of correlation from blue (positive) to red (negative). *ABA* abscisic acid, *SA* salicylic acid, *OPDA* 12-*oxo*-phytodienoic acid, *JA* jasmonic acid, *JA-Ile* jasmonoyl isoleucine, *iPA* isopentenyl adenosine, *2iP* 2-isopentenyl adenine, *ZR* zeatin riboside, *t-Z* *trans*-zeatin, *IAA* indole-3-acetic acid

Together with ABA, jasmonates are key hormones in the response to water stress, mainly aiding ABA in stomatal closure regulation (Savchenko et al. 2014). In the present study, water stress strongly reduced JA and JA-Ile root concentrations, while contents of the jasmonate precursor OPDA displayed a mycorrhiza-dependent response: OPDA increased in unstressed mycorrhizal plants in comparison to non-mycorrhizal individuals, and it decreased when water stress was applied. In addition, mycorrhization also enhanced JA contents in leaves, especially in stressed plants. This decrease in root OPDA and increase of leaf JA in mycorrhizal plants, particularly in water-stressed individuals, may be suggesting a mycorrhiza-enhanced active transport of the former from the roots to the leaves, where it may be converted into JA. This root-to-shoot transport of jasmonates upon water stress has been previously observed in tomato (De Ollas et al. 2018), and mycorrhization may trigger it as an acclimation signal in front of water stress, as suggested recently in *T. repens* (Fresno et al. 2023). Spearman correlation analyses between hormone and nutrient concentrations revealed a significant positive correlation (rho > 0.5, *P* < 0.001) between OPDA and two metals: Cu and Fe (Fig. 9). Both metals have been directly related to higher lipid peroxidation rates (Sandman and Böger 1980; Fang et al. 2001). This could imply that the enhanced absorption of Cu and other metals provided by AMF may be indirectly increasing OPDA production through an enhancement of lipid peroxidation. Nevertheless, more research should be made to clarify this hypothesis. Finally, it has been reported that AMF, as well as other beneficial microorganisms, partially suppress plant basal immune responses to enhance their colonization process (reviewed by Nishad et al. 2020). This may explain the lower levels of SA in mycorrhizal tomato roots, and it could imply a reduced capacity of the plant to defend from root pathogens. However, it has also been described that mycorrhizas can induce the expression of *PATHOGENESIS RELATED (PR)* defense genes in an SA-independent manner (Campos-Soriano et al. 2010; Campos-Soriano and San Segundo 2011), which could compensate AMF-induced basal immunity suppression.

Growth hormone levels were also influenced by water stress, leading, for example, to a systemic drop in cytokinin concentrations. The root system was the most affected organ, with significant reductions in both active cytokinins (2iP and *t*-Z) and their riboside precursors (iPA and *t*-ZR). This might be explained by the high relevance of this organ on the regulation of the systemic cytokinin dynamics. On the one hand, roots are the primary source of the precursor *t*-ZR, which is transported to the shoot via xylem vessels and transformed into active *t*-Z. On the other hand, shoot-synthesized iP-type cytokinins are transported to the roots, where they regulate the root system architecture (Hirose et al. 2008; Kudo et al. 2010; Sakakibara 2021). In the case of leaves, *t*-ZR transformation into *t*-Z was blocked by water stress, leading to an accumulation of the precursor and a drop of the active form that may explain the reduced shoot growth of water-stressed plants. Contrarily, the transformation of iPA into 2iP was reinforced upon water stress and mycorrhization, leading to lower precursor levels and similar 2iP concentrations to those of non-stressed individuals. In contrast to leaves, water stress decreased 2iP accumulation in underdeveloped fruits, while *t*-Z remained completely unaltered. These results point out that, apparently, the biological function and relevance of each cytokinin type in plant responses to water stress may vary depending on the plant organ, except in roots, where both types were actively modulated. On the other hand, the reduction in 2iP and IAA concentrations in underdeveloped and intermediate fruits of water-stressed plants, respectively, suggest that if the experiment had been carried out for a longer period of time, a drop in tomato production would have been observed, as a lack of both hormones lead to smaller and lighter tomato fruits (Matsuo et al. 2012; Gan et al. 2022). Finally, in contrast to the previous studies where mycorrhization improved cytokinin status (Adolfsson et al. 2017; Fresno et al. 2023), *R. irregulare* barely influenced cytokinin concentrations in the present study.

In addition to phytohormones, water deficit also altered the plant nutritional status. As previously described, water stress provoked a general nutrient starvation, although with some organ-specific variations (He and Dijkstra 2014). Water stress led to lower N content and higher C/N ratios in leaves and fruits, lower concentrations of micronutrients like Cu and Mn, and reduced P uptake in the roots. Mycorrhization, however, improved N, Cu and Zn contents in different tissues, including fruits, and alleviated P deficiency in roots upon water deficit. Correlation analyses revealed strong positive and significant correlations between some nutrients and cytokinins, particularly from the iP-type. Even though the influence of nutrients (mainly N, S and P) in cytokinin biosynthesis is well established and described, it was surprising that nutrient contents were more correlated with the precursor (iPA and *t*-ZR) than with the active forms (Fig. 9). Cytokinin ribosides are generally involved in long-distance transport within the plant, being *t*-ZR and iPA more abundant than their free forms in the xylem and phloem sap, respectively. Besides, they have been proven to be even more responsive to N fertilization than active forms (Osugi et al. 2017; Abualia et al. 2022), which highlight their role in plant response to fertilization. Thus, our results suggest that water stress-induced nutrient deficit could be altering systemic cytokinin transport by modulating cytokinin riboside precursor levels, which could eventually influence plant growth. However, more thorough research should be performed to clarify the mechanisms underlying this potential regulation.

Finally, water stress induced a systemic decrease in K, particularly remarkable in roots and fruits. Contrarily, Na concentrations were higher in leaf and fruits. As a result, Na/K ratios increased in roots, leaves, underdeveloped and intermediate fruits, but not in ripe fruits. Even though high Na concentrations may be toxic for the plant, in this experiment the levels reached were far below those observed in other experiments where salinity stress induced Na toxicity (Gharbi et al. 2017; Zhang et al. 2022b). Thus, the increase in Na may be playing another role in water stress response. For example, some studies have reported that Na may partially substitute K in its function as an osmolyte when the latter is lacking, such in the case of water stress (Erel et al. 2014; Nieves-Cordones et al. 2019; Thorne and Maathuis 2022). Nevertheless, a more detailed study should be performed to test this possibility. Finally, Ca response to water stress followed a tissue-specific pattern: it increased in roots, while it was slightly reduced in fruits. Ca is mainly transported to the shoot apoplastically, relying on evapotranspiration for a correct transport, which may be interrupted upon water stress due to stomata closure (Gilliham et al. 2011). Therefore, it is likely that a reduction in stomatal conductance may trigger this differential tissue-related Ca accumulation, which warrants further investigation.

To conclude, hormonal and nutrient responses to water stress followed a tissue-specific pattern, with some responses occurring systemically (e.g., ABA accumulation). The root system was revealed as a hormonal regulatory hub in front of water stress, being particularly relevant in the regulation of jasmonate and cytokinin responses. Leaves and fruits displayed as well specific hormonal responses in response to water deficit, despite this differential response appeared to become progressively less apparent with the advance of fruit ripening. Nutrient content strongly correlated with cytokinin ribosides levels, suggesting that the plant nutrient status may affect cytokinin biosynthesis and transport in the plant. Finally, mycorrhization improved fruit production, probably by enhancing nutrient absorption, and it triggered a specific OPDA and JA response that could imply a differential root-to-shoot jasmonate signaling.

### *Author contribution statement*

DHF and SMB conceived and designed the research; DHF conducted the experiments; DHF and SMB wrote and edited the manuscript.

## Supplementary Information

Below is the link to the electronic supplementary material.

Supplementary file1 (PDF 2181 kb)

## Data Availability

Data will be made available upon reasonable request.

## Abbreviations

AMF: Arbuscular mycorrhizal fungi
dad: Days after drought
JA: Jasmonic acid
JA-Ile: Jasmonoyl isoleucine
OPDA: 12-*Oxo*-Phytodienoic acid
iPA: Isopentenyl adenosine
2iP: 2-Isopentenyl adenine
*t*-Z: *trans*-Zeatin
*t-*ZR: *trans*-Zeatin riboside
RWC: Relative water content
SA: Salicylic acid
