# Supplementary material for: Organ-specific responses during acclimation of mycorrhizal and non-mycorrhizal tomato plants to a mild water stress reveal differential local and systemic hormonal and nutritional adjustments
Source: Planta. 2023 Jun 27;258(2):32. doi: 10.1007/s00425-023-04192-2 (PMC10300162; doi:10.1007/s00425-023-04192-2)
Supplement: Supplementary file 1 — Supplementary file1 (PDF 2181 kb) [file 425_2023_4192_MOESM1_ESM.pdf]

Organ-specific responses during acclimation of mycorrhizal and non-mycorrhizal tomato plants to a mild water stress reveal differential local and systemic hormonal and nutritional adjustments

David H. Fresno [1,2] and Sergi Munné-Bosch [1,2*]

*Correspondence: smunne@ub.edu

[a] Department of Evolutionary Biology, Ecology and Environmental Sciences, Faculty of Biology, University of Barcelona, Barcelona, Spain

[b] Institute of Nutrition and Food Safety (INSA), Faculty of Biology, University of Barcelona, Barcelona, Spain

Supplementary Information

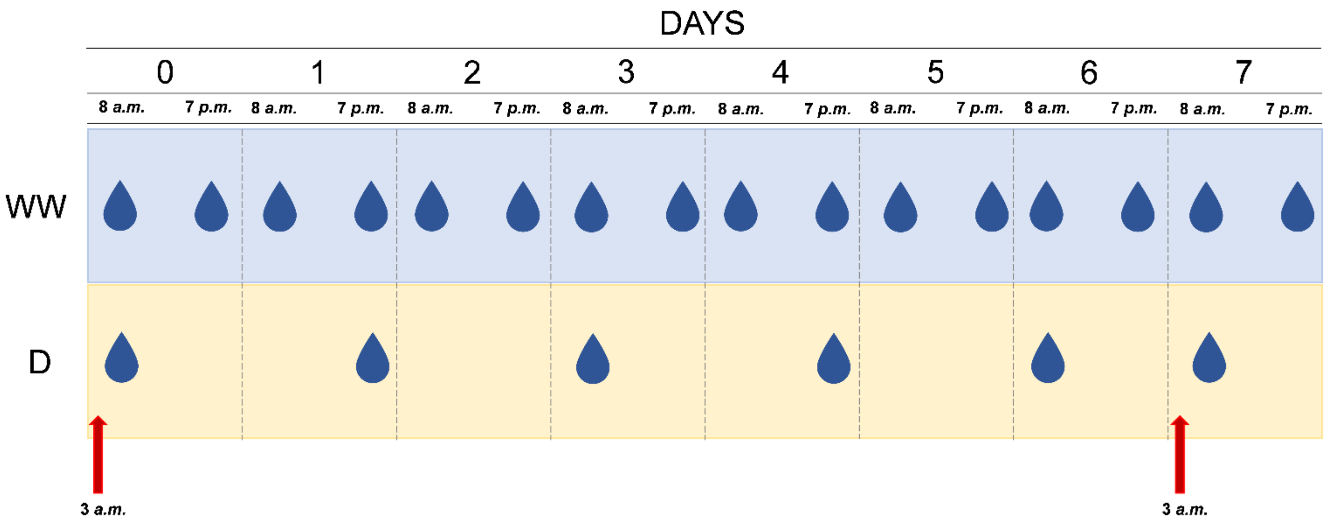

Suppl. Fig. S1. Irrigation pattern and sampling performance. Well-watered (WW) plants were irrigated twice a day at 8 *a.m.* and 7 *p.m.* using a drop irrigation system. Water-stressed (D) plants only received 1 out of every 3 irrigation events. Water drop symbols represent 1 irrigation event. Samplings (red arrows) were performed pre-dawn (3 *a.m.*) every 7 days. After each sampling, the irrigation pattern was restarted at 8 *a.m.* for another 7 days until the end of the experiment.

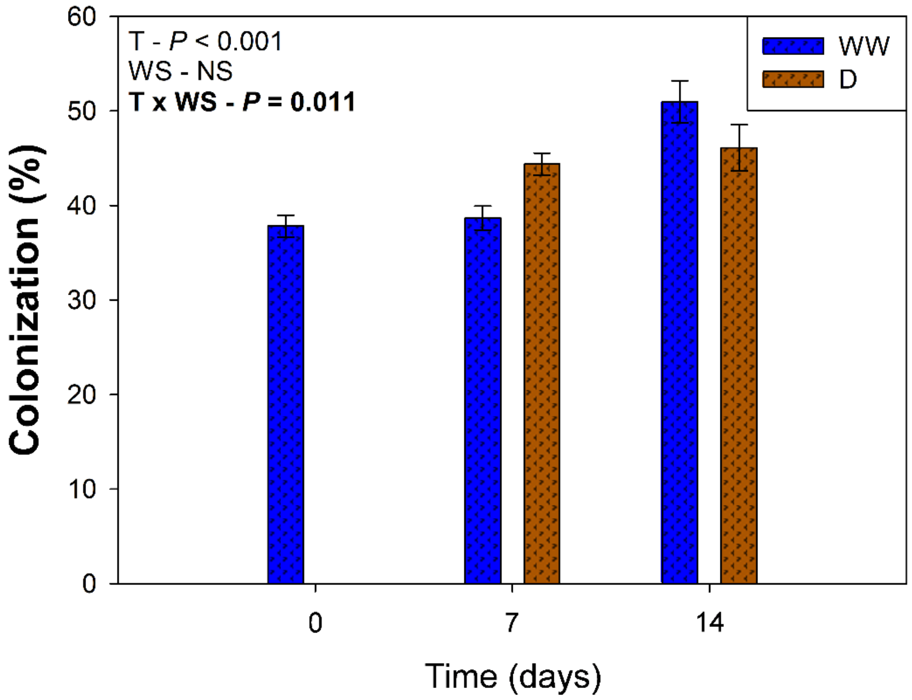

Suppl. Fig. S2. Mycorrhizal colonization of tomato plants inoculated with the arbuscular mycorrhizal fungus *Rhizoglomus irregulare* submitted to a well-watered (WW) or mild water stress (D). Measurements were taken every 7 days after the beginning of the water irrigation treatment. Data are means ± SE of n=5. Effects of 'Time' (T), 'Water Stress' (WS) and their interaction were evaluated by performing a two-way ANOVA. Factors with a statistical significance ($P < 0.05$) are highlighted in bold letters or marked as NS when non-significant ($P > 0.05$).

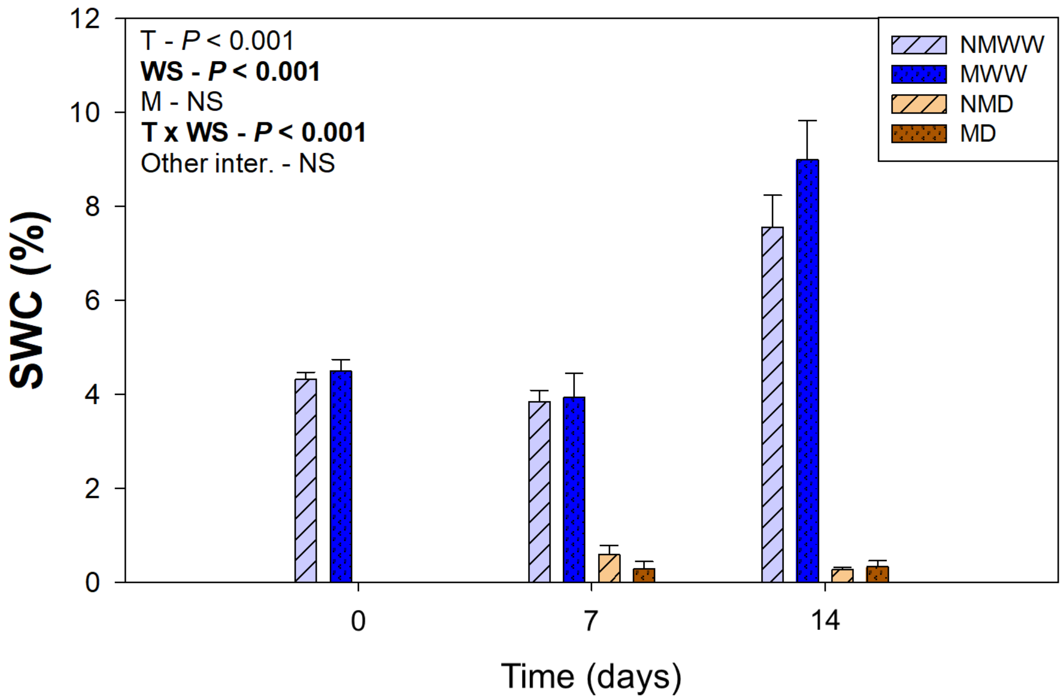

Suppl. Fig. S3. Soil water content (SWC) in pots of well-watered (WW) and water-stressed (D) tomato plants colonized (M) or not (NM) by the arbuscular mycorrhizal fungus *Rhizoglomus irregulare*. Measurements were taken every 7 days after the beginning of the water irrigation treatment. Data are means ± SE of n=5. Effects of 'Time' (T), 'Water Stress' (WS), 'Mycorrhization' (M), and all their interactions were evaluated by performing a three-way ANOVA. Factors with a statistical significance (P < 0.05) are highlighted in bold letters or marked as NS when non-significant (P > 0.05).

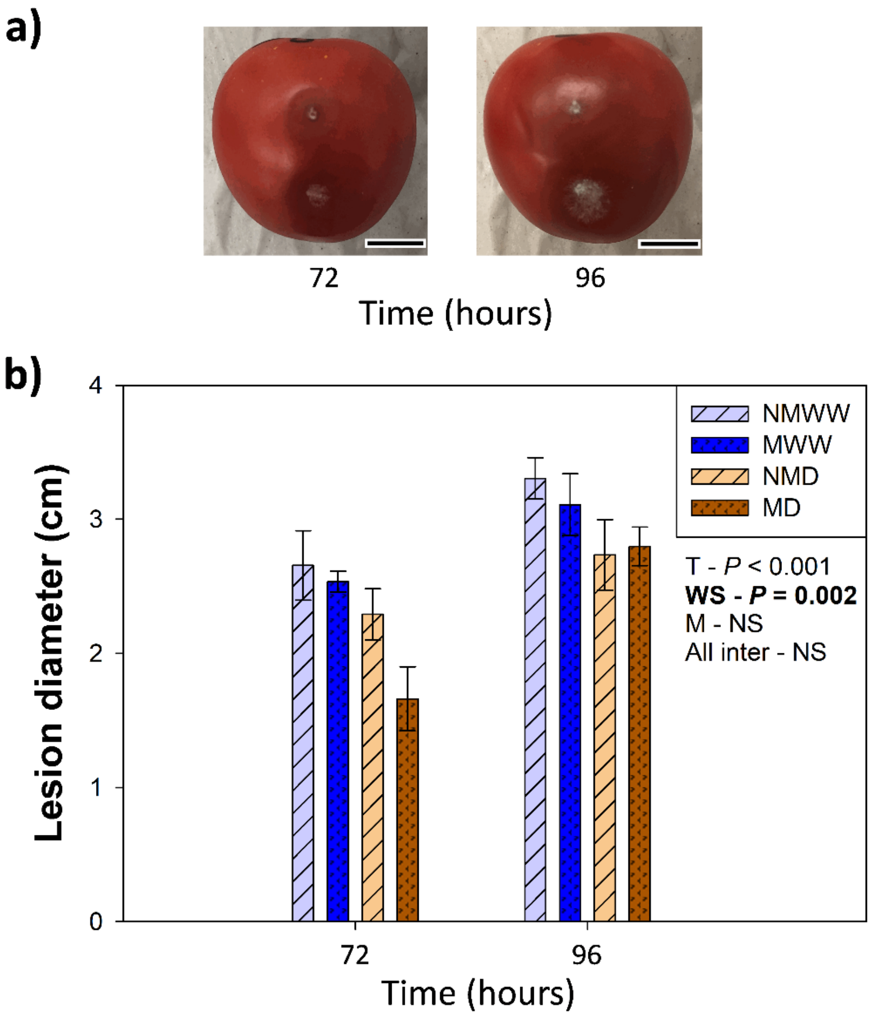

Suppl. Fig. S4. Mycorrhization does not improve tomato fruit resistance to *Botrytis cinerea*. **(a)** Ripe tomato fruits 72 and 96 hours after the inoculation with *B. cinerea.* **(b)** Mean lesion diameter caused by *B. cinerea* in ripe tomatoes coming from mycorrhizal (M) and non-mycorrhizal (NM) plants under a well-watered (WW) or a mild water stress (D) regime. Data are means ± SE of n=3, with each tomato fruit having two infection points. Effects of 'Time' (T), 'Mycorrhization' (M), 'Water Stress' (WS) and all the interactions were evaluated by performing a three-way ANOVA. Scale bar corresponds to 1 cm. Factors with a statistical significance ($P < 0.05$) are highlighted in bold letters or marked as NS when non-significant ($P > 0.05$).

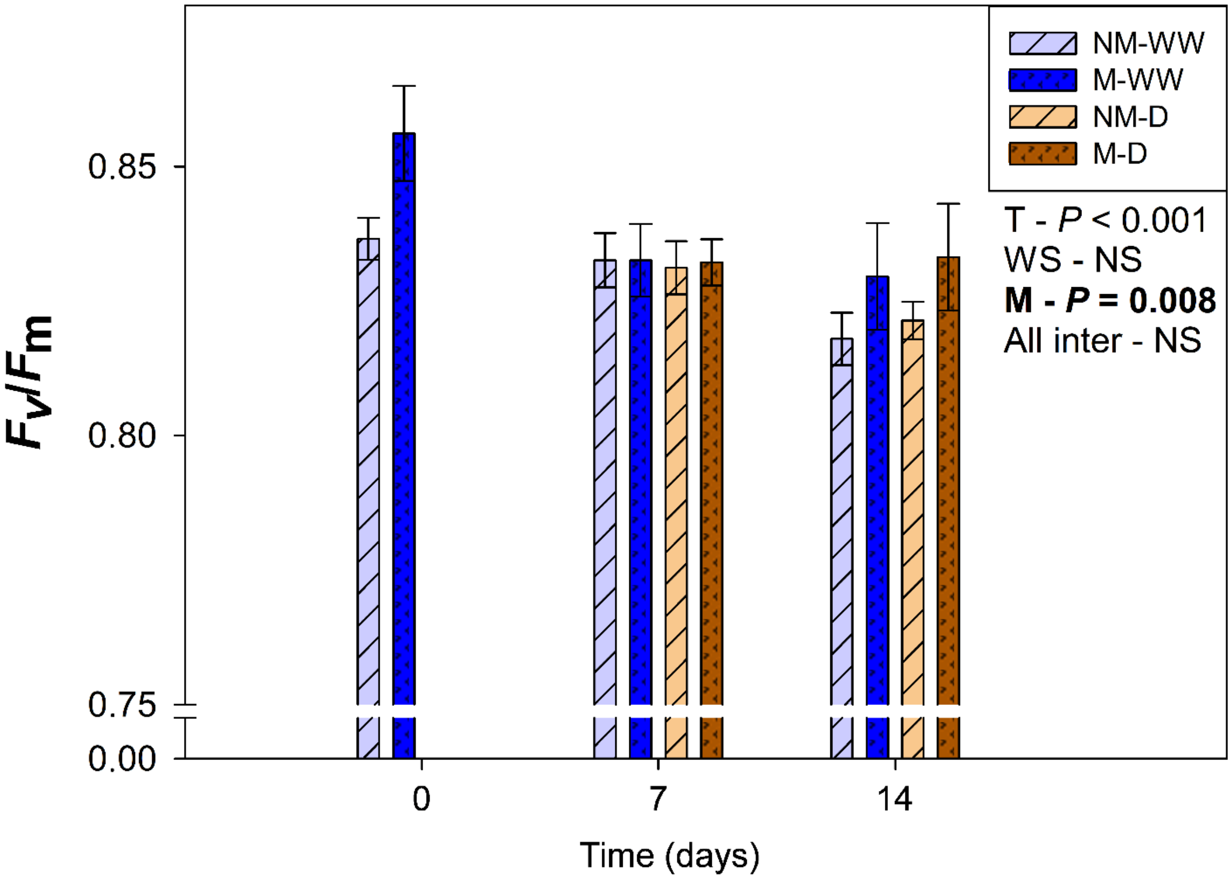

Suppl. Fig. S5. Maximal photochemical efficiency of photosystem II (*F*v/*F*m) of tomato plants submitted to a well-watered (WW) or mild water stress (D) treatment in the presence (M) or absence (NM) of the arbuscular mycorrhizal fungus *Rhizoglomus irregulare*. Measurements were taken every 7 days after the beginning of the water irrigation treatment. Data are means ± SE of n=5. Effects of 'Time' (T), 'Water Stress' (WS), 'Mycorrhization' (M), and all their interactions were evaluated by performing a three-way ANOVA. Factors with a statistical significance (P < 0.05) are highlighted in bold letters or marked as NS when non-significant (P > 0.05).

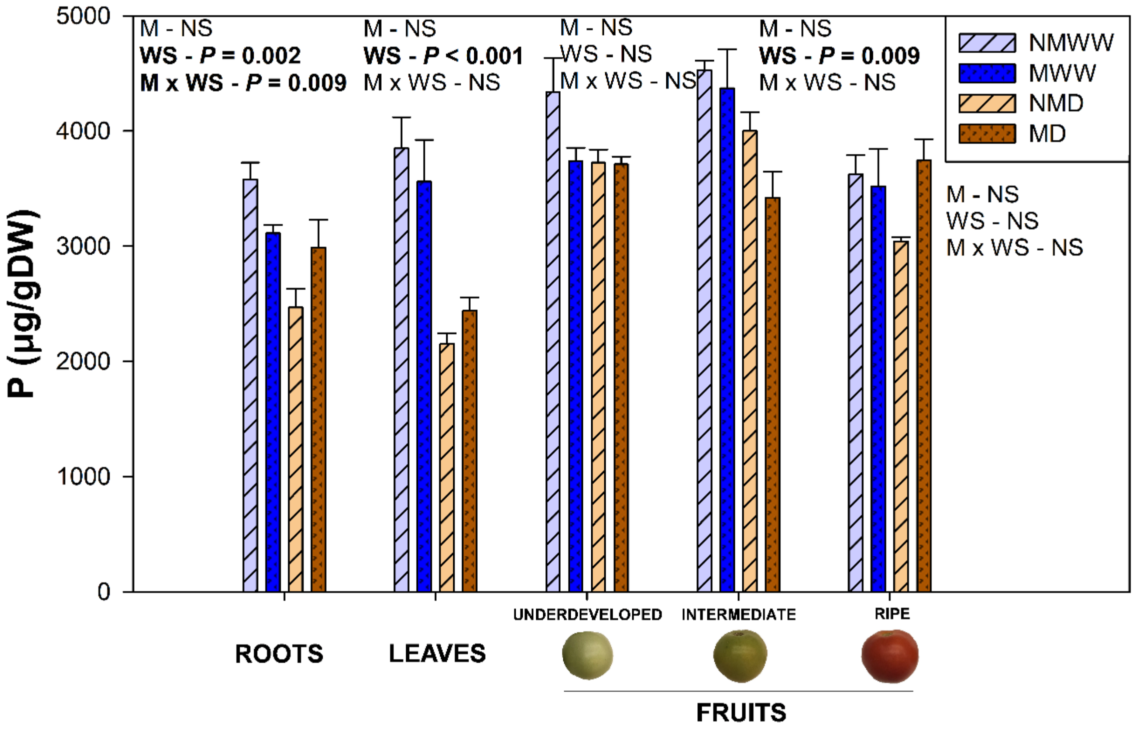

Suppl. Fig. S6. Mycorrhization by *Rhizoglomus irregulare* alleviates water stress-driven phosphorus (P) starvation. Endogenous P concentration 14 days after the beginning of a mild water stress treatment in roots, leaves and different developmental tomato fruit stages in well-watered (WW) and water stress-treated (D) plants, inoculated (M) or not (NM) with the arbuscular mycorrhizal fungus *R. irregulare*, are represented. Data are means ± SE of n=5 plants for roots and leaves, and of n=3 for each developmental fruit stage. Effects of 'Mycorrhization' (M), 'Water Stress' (WS) and their interaction (MxWS) were evaluated by performing a two-way ANOVA for each tissue. Factors with a statistical significance (P < 0.05) are highlighted in bold letters or marked as NS when non-significant (P > 0.05).

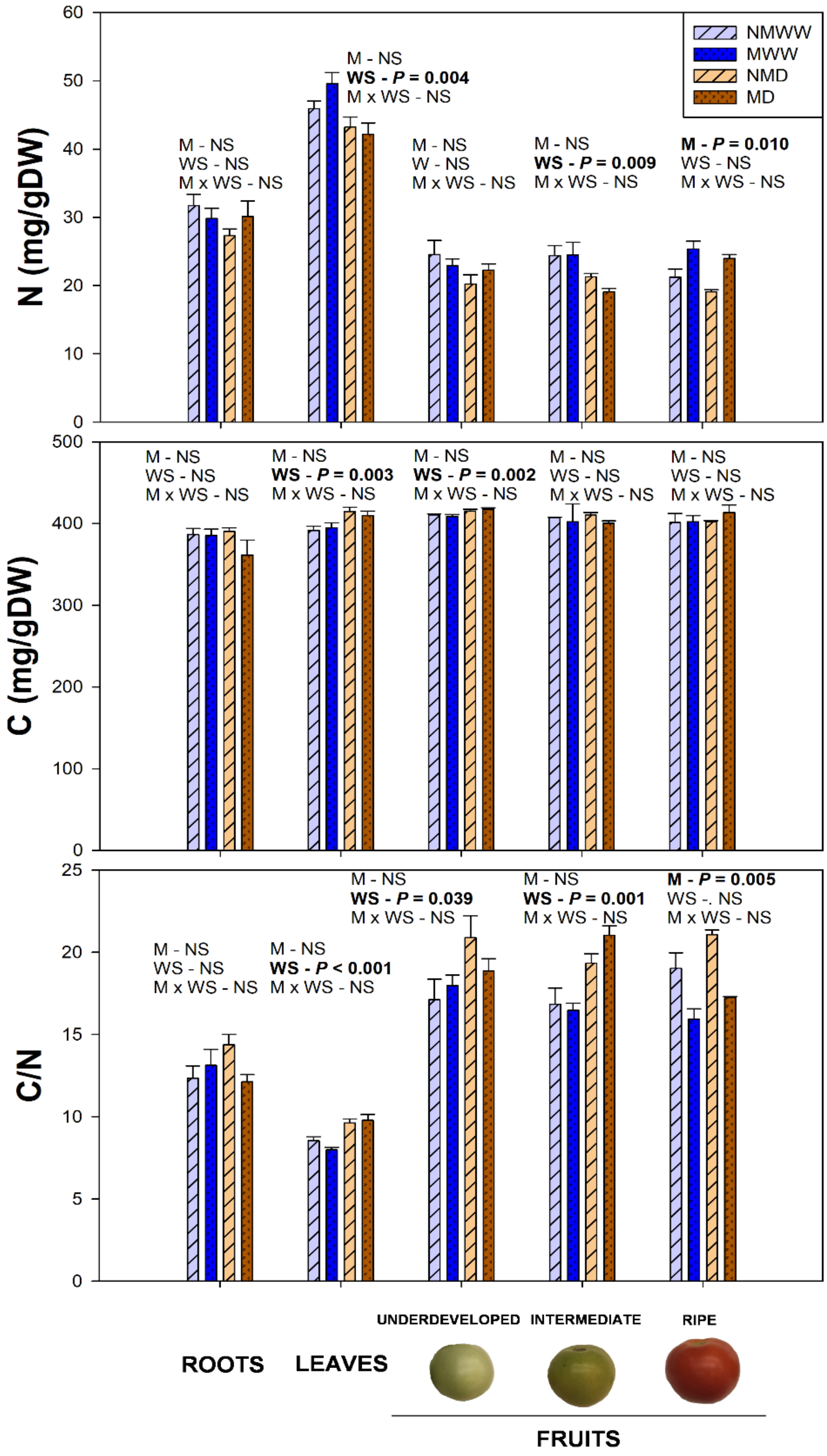

Suppl. Fig. S7. Carbon (C) and nitrogen (N) content is affected by a mild water stress and mycorrhization in different organs in tomato. Endogenous C, N and C/N ratio 14 days after the beginning of a mild water stress treatment in roots, leaves and different developmental tomato fruit stages in well-watered (WW) and water stress-treated (D) plants, inoculated (M) or not (NM) with the arbuscular mycorrhizal fungus *R. irregulare*, are represented. Data are means ± SE of n=5 plants for roots and leaves, and of n=3 for each developmental fruit stage. Effects of 'Mycorrhization' (M), 'Water Stress' (WS) and their interaction (MxWS) were evaluated by performing a two-way ANOVA for each tissue. Factors with a statistical significance ($P < 0.05$) are highlighted in bold letters or marked as NS when non-significant ($P > 0.05$).

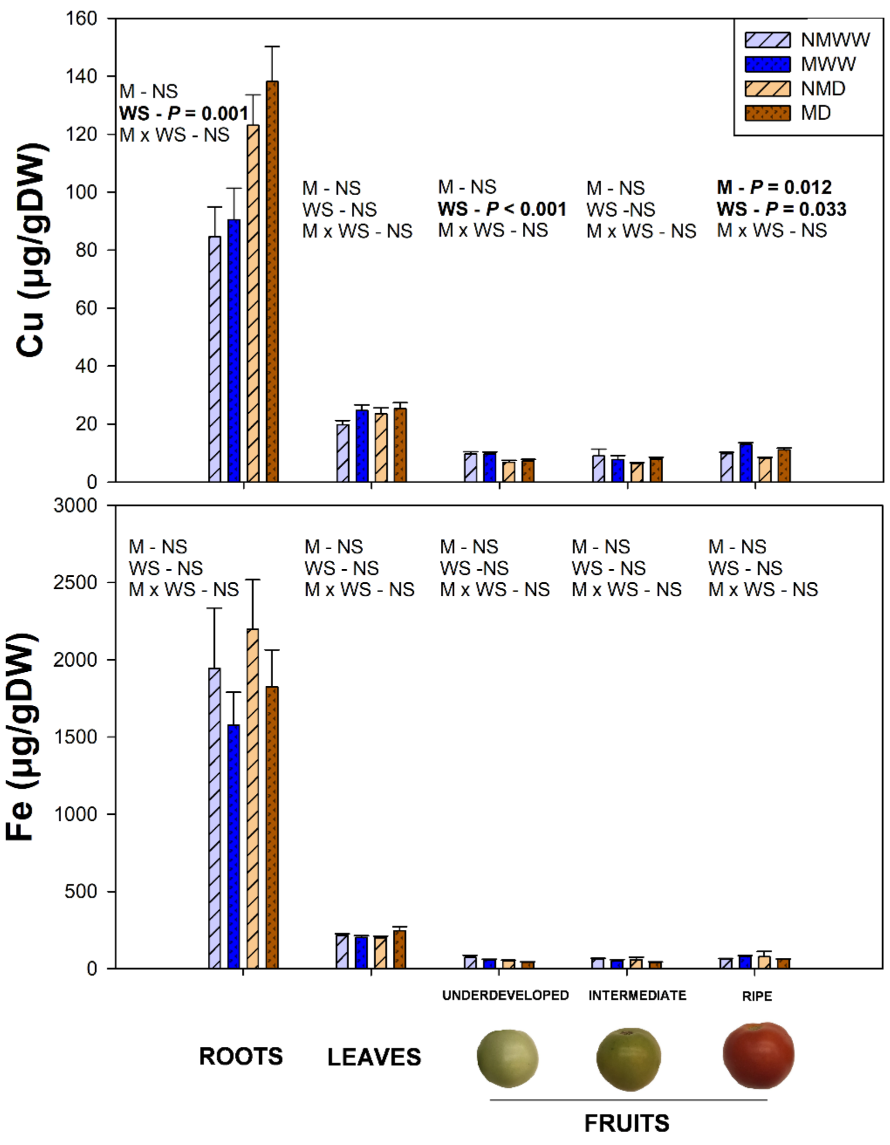

Suppl. Fig. S8. A mild water stress and mycorrhization have significant effects on copper (Cu) concentration in tomato. Endogenous Cu and iron (Fe) concentration 14 days after the beginning of a mild water stress treatment in roots, leaves and different developmental tomato fruit stages in well-watered (WW) and water stress-treated (D) plants, inoculated (M) or not (NM) with the arbuscular mycorrhizal fungus *R. irregulare*, are represented. Data are means ± SE of n=5 plants for roots and leaves, and of n=3 for each developmental fruit stage measured. Effects of 'Mycorrhization' (M), 'Water Stress' (WS) and their interaction (MxWS) were evaluated by performing a two-way ANOVA for each tissue. Factors with a statistical significance ($P < 0.05$) are highlighted in bold letters or marked as NS when non-significant ($P > 0.05$).

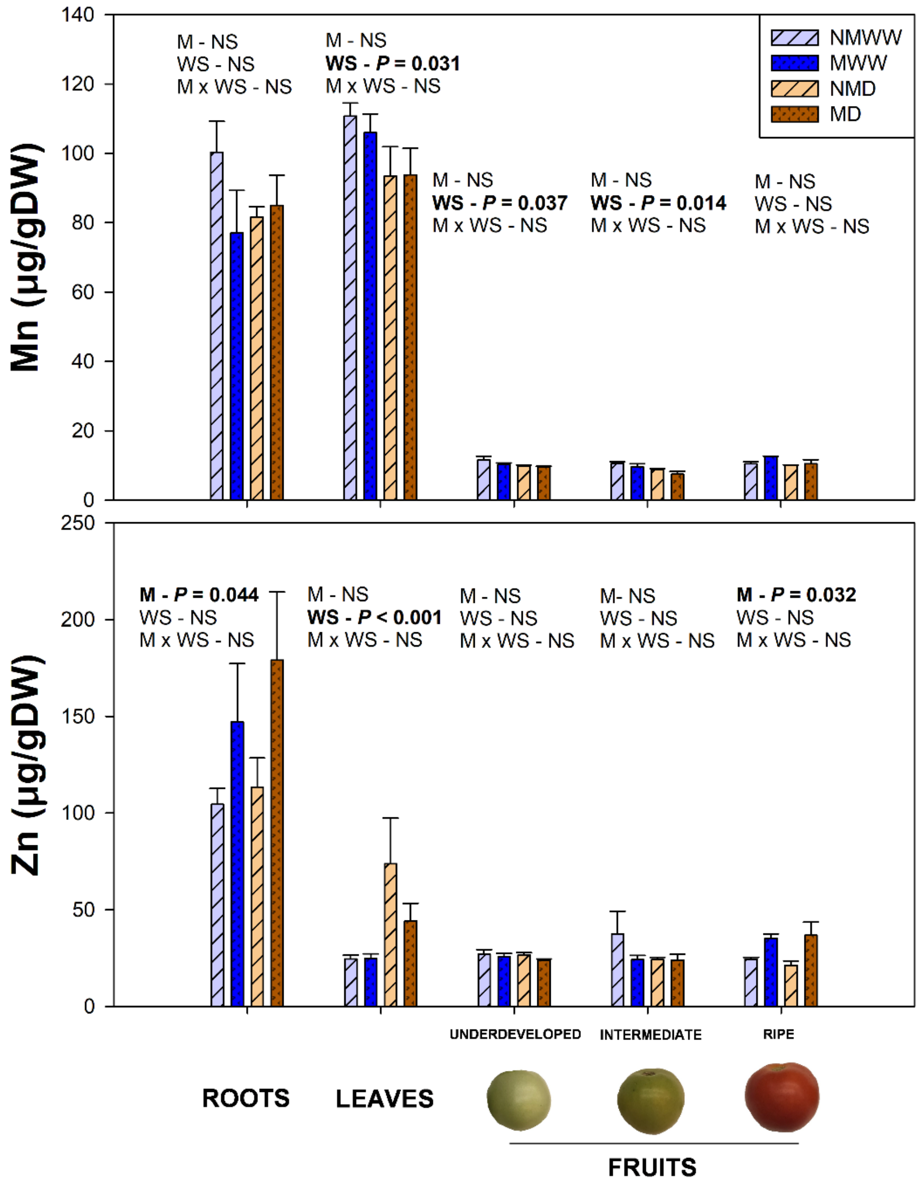

Suppl. Fig. S9. Mycorrhization improves zinc (Zn) contents in different organs in tomato plants. Endogenous manganese (Mn) and Zn concentration 14 days after the beginning of a mild water stress treatment in roots, leaves and different developmental tomato fruit stages in well-watered (WW) and water stress-treated (D) plants, inoculated (M) or not (NM) with the arbuscular mycorrhizal fungus *R. irregulare*, are represented. Data are means ± SE of n=5 plants for roots and leaves, and of n=3 for each developmental fruit stage measured. Effects of 'Mycorrhization' (M), 'Water Stress' (WS) and their interaction (MxWS) were evaluated by performing a two-way ANOVA for each tissue. Factors with a statistical significance (P < 0.05) are highlighted in bold letters or marked as NS when non-significant (P > 0.05).

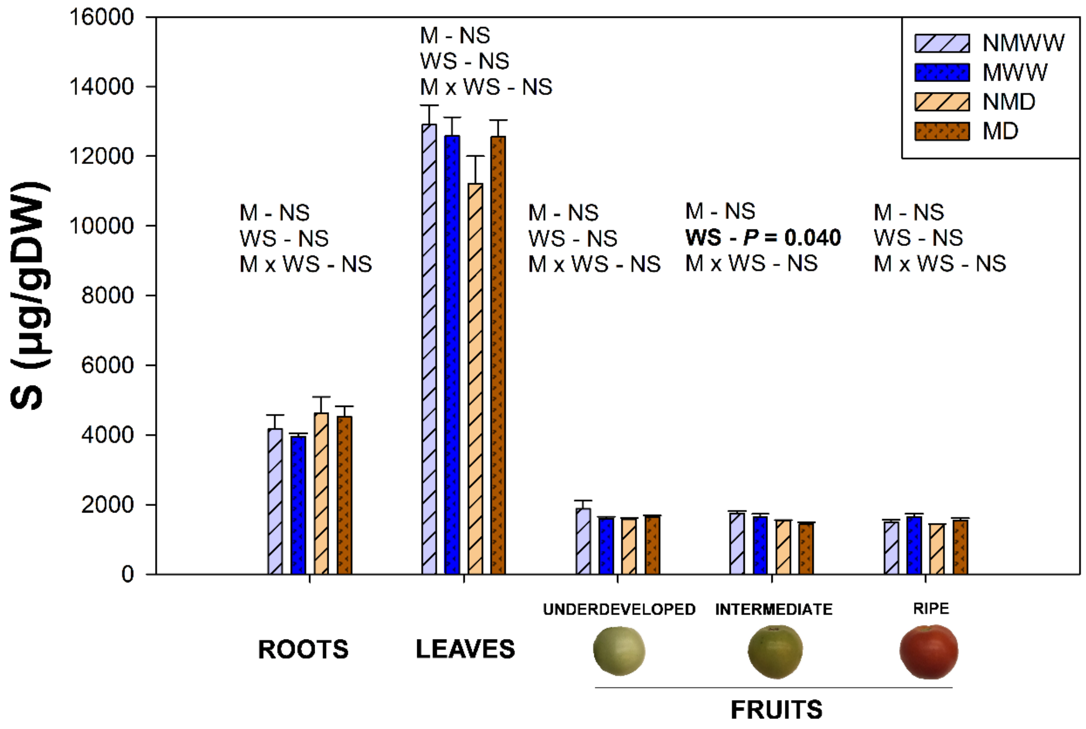

Suppl. Fig. S10. Sulfur (S) concentration is only affected by a mild water stress in intermediate fruits. Endogenous S concentration 14 days after the beginning of a mild water stress treatment in roots, leaves and different developmental tomato fruit stages in well-watered (WW) and water stress-treated (D) plants, inoculated (M) or not (NM) with the arbuscular mycorrhizal fungus *R. irregulare*, are represented. Data are means ± SE of n=5 plants for roots and leaves, and of n=3 for each developmental fruit stage measured. Effects of 'Mycorrhization' (M), 'Water Stress' (WS) and their interaction (MxWS) were evaluated by performing a two-way ANOVA for each tissue. Factors with a statistical significance (P < 0.05) are highlighted in bold letters or marked as NS when non-significant (P > 0.05).

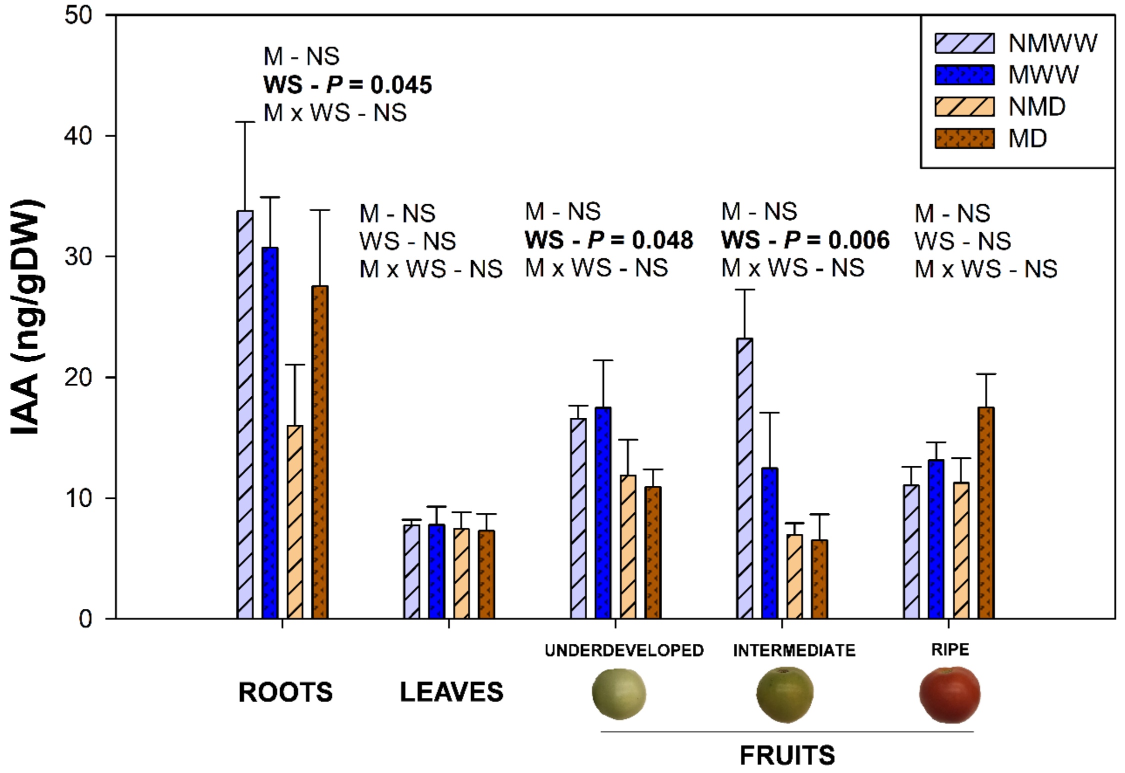

Suppl. Fig. S11. Endogenous content of the auxin indole acetic acid (IAA) after a 14 day-long mild water stress treatment is reduced in roots, leaves and different developmental tomato fruit stages in well-watered (WW) and water stress-treated (D) plants, independently on the presence (M) or absence (NM) of the arbuscular mycorrhizal fungus *Rhizoglomus. irregulare*. Data are means ± SE of n=5 plants for roots and leaves, and of n=3 for each developmental fruit stage measured. Effects of 'Mycorrhization' (M), 'Water Stress' (WS) and their interaction (MxWS) were evaluated by performing a two-way ANOVA for each tissue. Factors with a statistical significance ($P < 0.05$) are highlighted in bold letters or marked as NS when non-significant ($P > 0.05$).

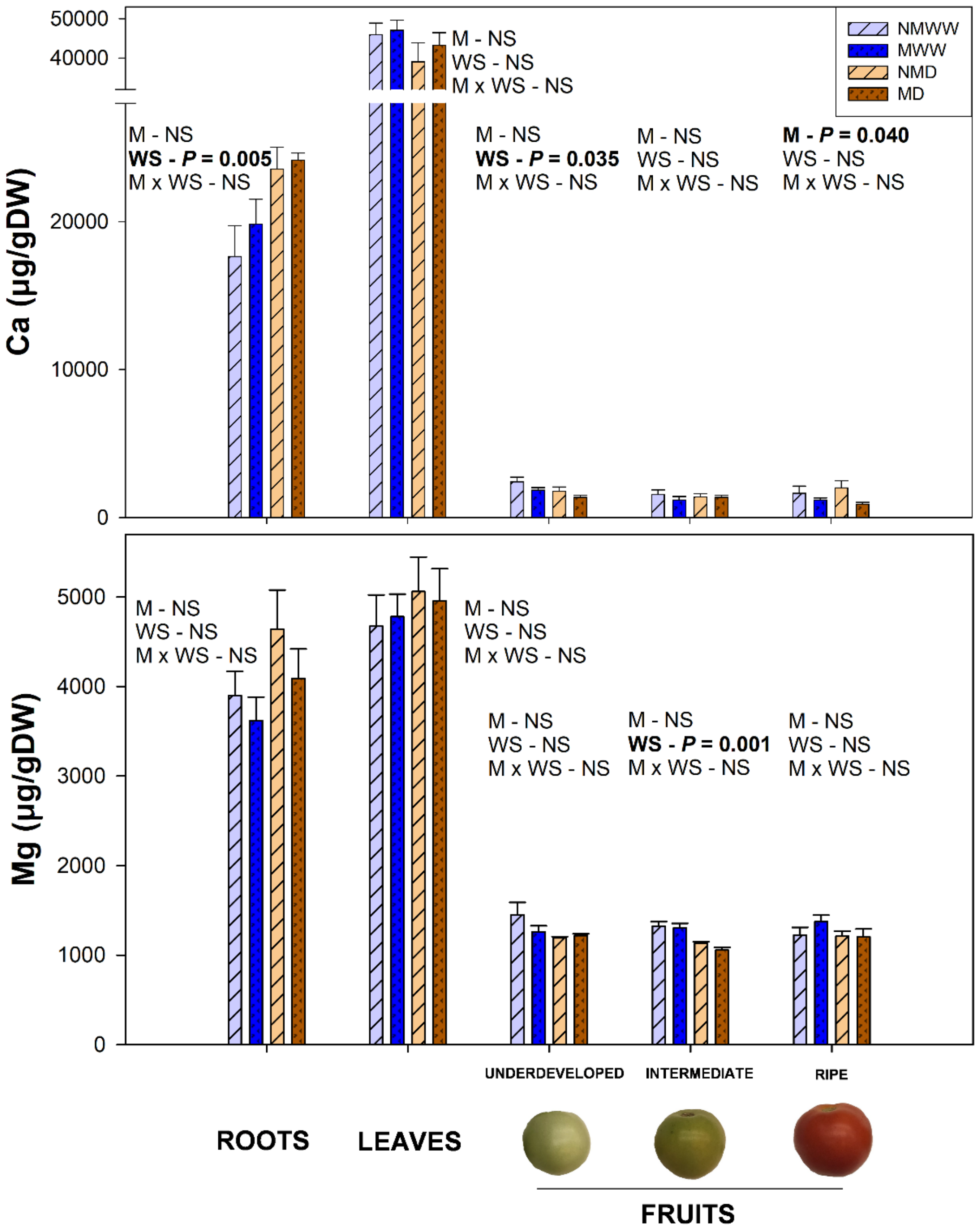

Suppl. Fig. S12. Endogenous content of calcium (Ca) and magnesium (Mg) in roots, leaves and different developmental tomato fruit stages in well-watered (WW) and water stress-treated (D) plants, in the presence (M) or absence (NM) of the arbuscular mycorrhizal fungus *Rhizoglomus. irregulare*. Data are means ± SE of n=5 plants for roots and leaves, and of n=3 for each developmental fruit stage measured 14 days after the beginning of the mild water stress treatment. Effects of 'Mycorrhization' (M), 'Water Stress' (WS) and their interaction (MxWS) were evaluated by performing a two-way ANOVA for each tissue. Factors with a statistical significance ($P < 0.05$) are highlighted in bold letters or marked as NS when non-significant ($P > 0.05$).

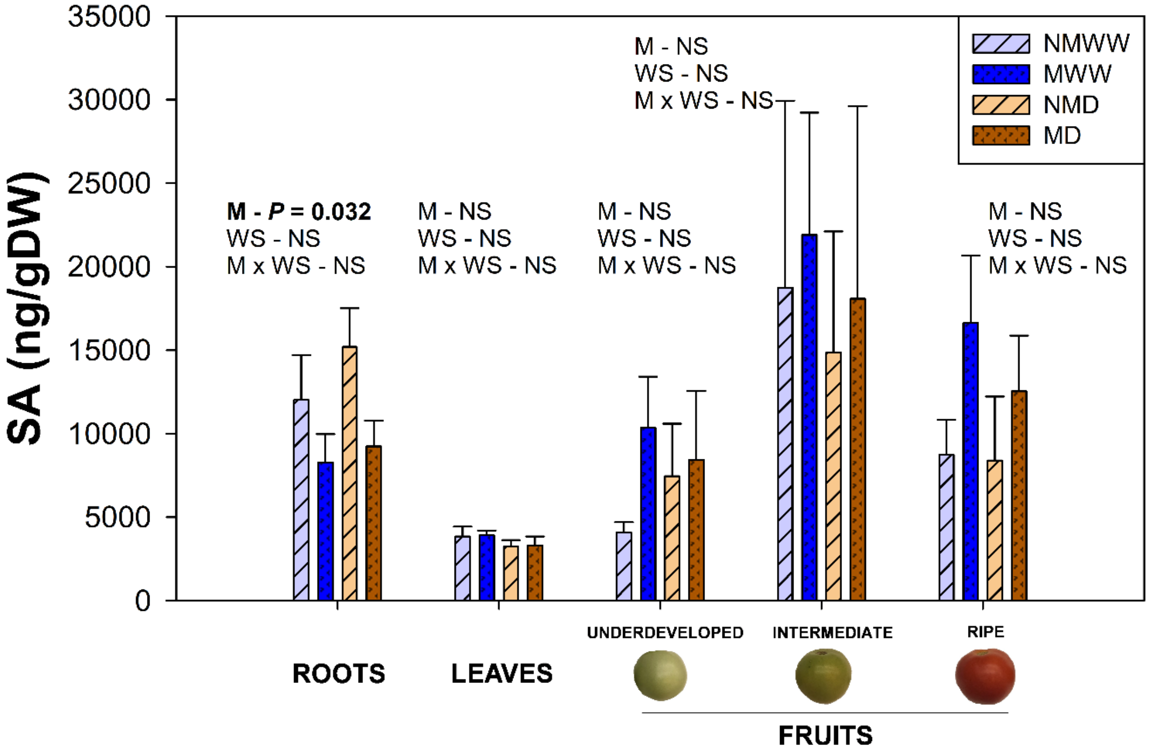

Suppl. Fig. S13. Salicylic acid (SA) is affected by mycorrhization exclusively in roots. Endogenous SA concentration 14 days after the beginning of a mild water stress treatment in roots, leaves and different developmental tomato fruit stages in well-watered (WW) and water stress-treated (D) plants, inoculated (M) or not (NM) with the arbuscular mycorrhizal fungus *R. irregulare*, are represented. Data are means ± SE of n=5 plants for roots and leaves, and of n=3 for each developmental fruit stage. Effects of ‘Mycorrhization’ (M), ‘Water Stress’ (WS) and their interaction (MxWS) were evaluated by performing a two-way ANOVA for each tissue. Factors with a statistical significance ($P < 0.05$) are highlighted in bold letters or marked as NS when non-significant ($P > 0.05$).
